# Free-Standing Boron-Doped Graphene Hydrogel Buckypaper Fabricated via Zinc-Plate Reduction for High-Performance Supercapacitors

**DOI:** 10.1021/acsami.6c00158

**Published:** 2026-03-26

**Authors:** Jia-Yu Ji, Jeremiah Hao Ran Huang, Shih-Wen Tseng, Yi-Wen Chen, Jhao-Rong Huang, I-Wen Peter Chen

**Affiliations:** † Department of Chemistry, 34912 National Cheng Kung University, No. 1, University Road, East District, Tainan 701, Taiwan; ‡ Interdisciplinary Research Center on Material and Medicinal Chemistry, National Cheng Kung University, No. 1, University Road, East District, Tainan 701, Taiwan; § Core Facility Center, National Cheng Kung University, Tainan 701, Taiwan; ∥ Graduate Institute of Biomedical Sciences, 38019 China Medical University, Taichung 404, Taiwan

**Keywords:** metal plate reduction, boron-doped rGO, graphene-based material, supercapacitor

## Abstract

Graphene offers exceptional mechanical flexibility and superior electrical conductivity that are highly desirable for next-generation supercapacitors; however, its intrinsic strong π–π stacking interactions severely hinder energy-storage performance by blocking active redox sites and restricting ion-accessible pathways. To address these limitations, we developed a binder-free, free-standing, boron-doped graphene hydrogel buckypaper with a hierarchical architecture. In this study, a mixed suspension of thermally activated boron-doped expanded graphene (BT-rGO) and graphene oxide (GO) was integrated via a redox reaction using a Zn plate as a substrate, enabling the instantaneous fabrication of a free-standing BT-rGO/Zn-rGO hydrogel buckypaper. The boron-doped graphene framework exhibits enhanced wettability, facilitating efficient electrolyte ion transportation, while the heteroatom-induced uneven charge distribution modulates the local electronegativity and improves electroconductivity throughout the free-standing hydrogel buckypaper network. The electrochemically enhanced E-BT-rGO_3_/Zn-rGO_1_ hydrogel buckypaper (BT-rGO:GO mass ratio = 3:1) delivers a high specific capacitance of 443.63 F g^–1^ at 2 mA cm^–2^ and maintains 88% capacitance retention after 10,000 cycles at 50 mA cm^–2^. Moreover, a flexible solid-state supercapacitor assembled from the BT-rGO_3_/Zn-rGO_1_ hydrogel buckypaper achieves an energy density of 16.96 Wh kg^–1^ and a power density of 5.21 kW kg^–1^, demonstrating its practical applicability for powering LED lightbulbs. Hence, the binder-free strategy creates a mechanically robust, porous framework with fully accessible ion pathways, delivering a purely carbon-based, metal-oxide-free electrode platform with an exceptional electrochemical performance.

## Graphical abstract

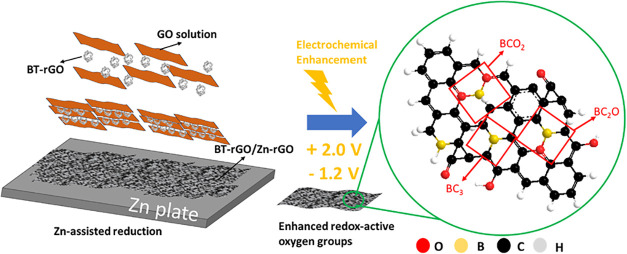

## 1 Introduction

The global demand for advanced energy-storage technologies has risen sharply with the rapid expansion of artificial intelligent (AI) data centers, which not only account for approximately 1.5% of global energy consumption but also require a reliable backup power system to mitigate power intermittency and ensure uninterrupted operation. Supercapacitors, in particular, have attracted significant interest owing to their theoretically ultrahigh power density, enabling rapid charge–discharge processes,^1,2^ and their exceptional cycling stability, often exceeding long life cycles.^3,4^ However, despite these advantages, practical supercapacitors suffer from intrinsically low energy density,^5,6^ arising from limited surface wettability, insufficient electrochemically active sites, and inadequate electrical conductivity. To address this challenge, substantial efforts have been focused on the development of earth-abundant, carbon-based materials with good surface wettability, high specific surface areas, and sufficient electrical conductivity. Graphene oxide (GO) initially emerged as a promising precursor owing to its theoretically large surface area and abundant chemical functionalities; however, the high concentration of oxygen-containing groups disrupts the π-conjugated carbon lattice, resulting in ultralow electrical conductivity and sluggish electron transport.^7,8^ Consequently, reducing GO to form reduced graphene oxide (rGO) has been shown to restore electrical conductivity^9,10^ and improve electrochemical performance.^11,12^ Nevertheless, the charge storage of pristine rGO largely relies on effective surface area, which was greatly restricted by intrinsic, strong π–π stacking interactions. To overcome this limitation, heteroatom doping has emerged as an effective and widely adopted strategy to reduce π–π stacking interactions while introducing redox-active sites.^13–17^ Incorporation of heteroatoms not only provides additional redox-active sites but also modulates the electronic structure, enhances surface reactivity, and collectively improves the energy-storage performance of graphene-related materials.

Incorporating heteroatoms into GO or rGO is an effective strategy to enhance charge-storage capability, primarily because dopants introduce reversible faradaic reactions that contribute pseudocapacitance (PC).^18–20^ Among various dopants, nitrogen-doped (N-doped) rGO exhibits increased capacitance due to the formation of pyridinic-N and pyrrolic-N sites,^21^ which provide abundant redox-active centers capable of rapid and reversible charge transfer. The strong electronegativity of nitrogen induces localized electron-rich regions that strongly adsorb electrolyte molecules.^22^ However, under high operating voltages, these polarized regions can trigger oxygen evolution or interfacial decomposition, narrowing the stable electrochemical potential window and limiting the charge-storage density.^23^ Phosphorus doping offers additional advantages through strong electron-donating behavior^24,25^ and the creation of highly active P-induced redox sites,^26,27^ particularly in acidic environments. Nevertheless, the relatively large atomic radius of P often induces lattice deformation, strain, and defect generation,^28,29^ while surface P–O species are likely to dissolve in aqueous or acidic electrolytes, compromising long-term interfacial stability.^30–32^ In contrast, boron doping provides a structurally compatible and electrochemically advantageous alternative due to its lower electronegativity relative to carbon. Boron acts as an electron acceptor, generating hole carriers and redistributing charge within the π-conjugated framework.^33,34^ This modulation enhances the electrical conductivity while preserving structural integrity. Moreover, a useful chemoinformatic perspective can be gained by considering bond dissociation energies (BDEs) of typical heteroatom–carbon bonds. Bond stability in carbon frameworks is often correlated with the intrinsic strength of the covalent bond between carbon and the dopant or heteroatom species since stronger bonds are generally less susceptible to cleavage under electrochemical stress or corrosive environments. According to established bond energy tables, the approximate mean bond energies of relevant single bonds illustrate this trend: C–N bonds (339.7–413.3 kJ mol^–1^)^35^ and C–P bonds (300.1–370.3 kJ mol^–1^)^36^ are systematically weaker than typical C–C bonds (around 418.4 kJ mol^–1^).^37^ By comparison, B–C bonds have typical bond energies (422.0–490.0 kJ mol^–1^)^38^ that are comparable to C–C and significantly stronger than C–N and C–P, indicating a generally more robust covalent interaction within carbon matrices. This suggests that B–C bonds formed upon boron incorporation are intrinsically more resistant to homolytic bond cleavage than many N- and P-related bonds in analogous doped systems, supporting the notion that boron dopants provide superior lattice resilience and interfacial stability. Additionally, B–C and B–O bonds suppress overoxidation at high anodic potentials, effectively broadening the electrochemical stability window,^39,40^ and increase surface polarity, improving wettability to facilitate electrolyte infiltration and accelerates ion transport.^41,42^

In this work, thermal activation of boric acid with commercial GO at 400 °C was employed to synthesize thermally activated boron-doped expanded graphene (BT-rGO). The relatively low annealing temperature allows effective boron doping while promoting the formation of stable B–C and B–O bonds and can maintain mild graphitization. GO was prepared via a modified Hummers’ method,^43,44^ with surface oxygen functional groups facilitating hydrogen-bonding interactions with B–O and C–O sites in BT-rGO.^45,46^ These interactions improve nanosheet dispersion, minimize aggregation, and enable the formation of a continuous BT-rGO/GO suspension suitable for hydrogel buckypaper fabrication. A free-standing BT-rGO/Zn-rGO hydrogel buckypaper was then produced via a metal-plate-assisted redox reaction, yielding a material with well-defined porosity, structural continuity, and mechanical robustness. The electrochemically enhanced E-BT-rGO_3_/Zn-rGO_1_ (BT-rGO/GO = 3:1) exhibits the highest electrochemical efficiency (86.89%) and a specific capacitance of 443.63 F g^–1^ at 2 mA cm^–2^ in 1 M H_2_SO_4_. The hydrogel buckypaper also shows excellent cycling durability, retaining 88% capacitance after 10,000 cycles at 50 mA cm^–2^. When assembled into a flexible all-solid-state supercapacitor, it achieves an energy density of 16.96 Wh kg^–1^ and a power density of 5.21 kW kg^–1^. These results demonstrate that the combined strategy of controlled boron doping, spontaneous metal plate reduction, and electrochemical activation provides a robust and generalizable approach to constructing high-energy density, high-power density, and mechanically robust heteroatom-doped graphene-based supercapacitors.

## 2 Experimental Section

### 2.1 Materials

Graphite flakes (natural, 325 mesh, 99.8%) were obtained from Alfa Aesar (Thermo Fisher Scientific, USA). Sodium nitrate (NaNO_3_, 99%, analytical grade) was sourced from Acros Organics. Hydrogen peroxide (H_2_O_2_, 30%, reagent special grade) and potassium permanganate (KMnO_4_, 99.3%, reagent special grade) were purchased from Showa Chemical Co., Ltd. Concentrated sulfuric acid (H_2_SO_4_, 96%) was acquired from Aencore. Hydrochloric acid (HCl, 37%) was purchased from Honeywell Fluka. Commercial graphene oxide powder (GO, model XFSG01) was provided by Jiangsu XFNANO Materials Tech Co., Ltd. Boric acid (H_3_BO_3_, ≥99.8%) was offered by Honeywell Fluka. Poly­(vinyl alcohol) (PVA, Mw 89,000–98,000, 99+% hydrolyzed) was supplied from Sigma-Aldrich. Ultrapure water (18.2 MΩ·cm) was applied in all solution preparations and following steps.

### 2.2 Synthesis of Graphene Oxide (GO) Solution

GO solution was synthesized via a modified Hummers method. First, 1.5 g of graphite and 0.75 g of NaNO_3_ were mixed with 37.5 mL of concentrated H_2_SO_4_. Subsequently, the mixture was stirred at 110 rpm and cooled for 20 min, followed by the gradual addition of 7.5 g of KMnO_4_, and then stirred at 220 rpm and 1.5 °C for 12 h with the top sealed with paraffin film. Then, 300 mL of ultrapure water and 4.5 mL of H_2_O_2_ were added dropwise to terminate the oxidation. The GO solution was centrifuged at 8000 rpm for 3 min to concentrate, then washed with 625 mL of HCl (5%) at 8000 rpm for 3 min, followed by 1500 mL of ultrapure water at 10,000 rpm for 10 min until the solution turned nearly neutral (pH = 6–7). Finally, the GO solution was treated with an ultrasonic bath for 5 min for dispersion (8 mg mL^–1^).

### 2.3 Preparation of Thermally Activated Boron-Doped Expanded Graphene (BT-rGO) and Thermally Reduced Graphene Oxide (T-rGO)

2 g portion of commercial GO powder and 0.5 g of H_3_BO_3_ were mixed in a mortar and ground to ensure uniform mixing. Figure S1 shows that the BT-rGO/Zn-rGO hydrogel buckypaper achieves the highest capacitance when the optimal H_3_BO_3_ weight is 0.5 g. Then, the powders were evenly spread in a rectangular alumina boat, covered with an alumina lid, and introduced into a tubular furnace under an Ar atmosphere. The powder was heated to 400 °C and maintained for 2 h, with a ramp time of 30 min and an Ar flow rate of 150 cc min^–1^. At about 200 °C, we found that the powder underwent thermal expansion because the release of gas led to a temporary pressure increase. After the heating process, the BT-rGO powder was stored in a serum bottle in an oven (55 °C). Similarly, thermally reduced graphene oxide (T-rGO) was prepared as a control but without boric acid.

### 2.4 Preparation of BT-rGO_*x*_/Zn-rGO_1_ Hydrogel Buckypaper

Preparation of the BT-rGO_*x*_/Zn-rGO_1_ hydrogel buckypaper had different ratios (BT-rGO_1_/Zn-rGO_1_, BT-rGO_2_/Zn-rGO_1_, and BT-rGO_3_/Zn-rGO_1_), and total weight remained at 240 mg. Specifically, 120, 160, and 180 mg of BT-rGO were mixed with 15, 10, and 7.5 mL of GO solution (8 mg mL^–1^) to attain each ratio, respectively. Each mixture was subjected to ultrasonic bath treatment for 40 min and then transferred to a Petri dish with a polished Zn plate for 2 h, where the BT-rGO and GO were reduced and self-assembled into BT-rGO_*x*_/Zn-rGO_1_ hydrogel buckypaper. The resulting material had a mass loading of 2.8 ± 0.2 mg cm^–2^. After the self-assembly process, the samples were washed with HCl (3.7 wt %) to remove residuals. Figure S2a shows that BT-rGO_3_/Zn-rGO_1_ can form a free-standing hydrogel buckypaper. Notably, when 200 mg of BT-rGO was mixed with 5 mL of GO solution (8 mg mL^–1^) to prepare BT-rGO_4_/Zn-rGO_1_ hydrogel buckypaper, the resulting material could not form a large-area hydrogel buckypaper, as shown in Figure S2b.

### 2.5 Preparation of E-BT-rGO_*x*_/Zn-rGO_1_ Hydrogel Buckypaper

The BT-rGO_3_/Zn-rGO_1_ hydrogel buckypaper was subsequently subjected to an electrochemical activation process by applying constant potentials of 2.0 V and −1.2 V for 180 s at each activation time. After the enhancement, the electrochemically enhanced hydrogel buckypaper was denoted as E-BT-rGO_3_/Zn-rGO_1_. Following the same activation procedure, the BT-rGO_2_/Zn-rGO_1_ and BT-rGO_1_/Zn-rGO_1_ hydrogel buckypapers were converted into their corresponding electrochemically enhanced samples, namely, E-BT-rGO_2_/Zn-rGO_1_ and E-BT-rGO_1_/Zn-rGO_1_, respectively.

### 2.6 Preparation of PVA/H_2_SO_4_ Gel

1.2 g portion of PVA was added to 12 mL of deionized water and stirred at 80 °C for 3 h until forming the transparent solution. Then, 0.70 mL of H_2_SO_4_ aqueous solution was slowly added and stirring was continued for 3 h. Next, the mixture was transfered into a plastic Petri dish and placed in a freezer at −16 °C for 3 h, followed by thawing at room temperature for 1 h. After three repeated cycles, the PVA/H_2_SO_4_ gel was obtained. The PVA/H_2_SO_4_ gel was stored in 1 M H_2_SO_4_ until use.

### 2.7 Preparation of Flexible Solid-State Supercapacitor

After electrochemical enhancement, the E-BT-rGO_3_/Zn-rGO_1_ hydrogel buckypaper (1 cm × 0.5 cm) was placed on hydrophobic conductive carbon cloth (1 cm × 3 cm) which served as the current collector. The PVA/H_2_SO_4_ gel (1.5 × 0.75 cm) was sandwiched between the two hydrogel buckypapers as both the electrolyte and separator. Additionally, the device was wrapped with cling film to maintain the device stability.

### 2.8 Characterizations

The material characterizations of BT-rGO powder and BT-rGO_*x*_/Zn-rGO_1_ hydrogel buckypapers were investigated by X-ray diffraction (Bruker D8 ADVANCE ECO), Raman spectroscopy (Horiba Jobin Yvon iHR550), Fourier-transform infrared spectroscopy (PerkinElmer Frontier FTIR94053, ATR mode), X-ray photoelectron spectroscopy (PHI VersaProbe 4), and Brunauer–Emmett–Teller (ASAP 2060 analyzer Micromeritics) analysis.

### 2.9 Electrochemical Measurement

The electrochemical performance was measured using cyclic voltammetry (CV), galvanostatic charge–discharge (GCD), and electrochemical impedance spectroscopy (EIS) on a multifunctional electrochemical workstation (CHI7279E, CH Instruments) at room temperature. A three-electrode system was used, where two BT-rGO_*x*_/Zn-rGO_1_ hydrogel buckypapers served as both working and counter electrodes. Filter paper served as a separator for the two hydrogel buckypapers, and the Pt foil acted as the current collector, forming a sandwich structure. The entire system was soaked in 1 M H_2_SO_4_ electrode along with an Ag/AgCl reference electrode. The gravimetric capacitance (*C*_wt_) of a single electrode was obtained from GCD curves by the eq 1

1 $$C_{\mathrm{wt}}\;\left({F\;g}^{-1}\right)=\frac{I\times \Delta t}{m\times U}$$

where *I* is the constant discharge current, Δ*t* is the discharge time, *m* is the mass loading of single electrode, and *U* is the voltage window.

The gravimetric energy density (*E*_wt_) and power density (*P*_wt_) of the device were calculated by eqs 2 and 3

2 $$E_{\mathrm{wt}}=\frac{C_{\mathrm{wt}}\times U^{2}}{8}$$

3 $$P_{\mathrm{wt}}=\frac{E_{\mathrm{wt}}\times 3600}{\Delta t}$$

## 3 Results and Discussion

Figure 1a shows the (002) diffraction peak of T-rGO and BT-rGO appear at around 25.0°, confirming the effective deoxygenation of GO to form rGO. In addition, the BT-rGO exhibits a full width at half-maximum (FWHM) of 3.6, which is significantly narrower than the 5.4 observed for T-rGO, indicating more uniform interlayer spacing associated with graphitic stacking in BT-rGO.^47^ In Figure 1b, FT-IR spectra of BT-rGO exhibit clear redshifts in the −OH stretching peak and the C–C (from 1216 to 1193 cm^–1^) stretching peaks compared with T-rGO. These shifts likely arise from changes in bond force constants induced by the formation of B–C (1418 cm^–1^). In addition, the appearance of the B–O vibration at 788 cm^–1^ suggests that boron incorporation redistributes the local electron density in BT-rGO, leading to a d electron-deficient nature that intercalates into the carbon lattice to induce local lattice distortion and modify surface chemical properties.^48^ This redistribution is consistent with the enhanced −OH peak features, indicating that the boron-rich regions have a higher affinity for oxygen-containing species, thereby increasing surface polarity and improving wettability. In Figure S3, the XPS spectra of T-rGO and BT-rGO exhibit two and three characteristic peaks corresponding to C 1s, O 1s, and B 1s signals, respectively. The atomic ratios of the O/C atoms in Table S1 are around 0.309 for T-rGO and 0.325 for BT-rGO, which correspond to the FT-IR spectra showing that boron has a higher affinity for oxygen functional groups. Figure 1c shows that the nitrogen adsorption–desorption isotherms reveal a pronounced decrease (from 405.67 to 110.39 m^2^ kg^–1^) in the overall adsorption capacity after boron doping, evidenced by the substantially suppressed adsorbed volume across the entire relative-pressure range. Correspondingly, the pore-size distribution curves in Figure 1d show a clear attenuation and narrowing of the micropore peak, indicating that boron incorporation leads to generation of fine pores (from 2.1 to 0.50 cm^3^ g^–1^), suggesting that boron incorporation alters the microstructure of rGO sheets.^49,50^ In Figure 1e, the Raman spectra reveal BT-rGO blue shifts in both the D band (from 1344.4 to 1346.1 cm^–1^) and G band (from 1573.1 to 1579.2 cm^–1^) compared with T-rGO, accompanied by an increased *I*_D_/*I*_G_ ratio (from 1.04 to 1.33). These changes indicate hole-type doping arising from the electron-deficient nature of boron atoms. The rise in *I*_D_/*I*_G_ highlights an increase in the defect density, particularly substitutional defects and edge distortions related to B–C bonding. Additionally, the G-band upshift indicates enhanced sp^2^ domain coupling and strengthens in-plane C–C bonding, while the D-band shift reflects lattice strain and local symmetry breaking caused by boron incorporation.^51^ These results confirm that boron doping leads to an electronically polarized, defect-enriched, and structurally reconstructed rGO framework.

**Figure 1:**
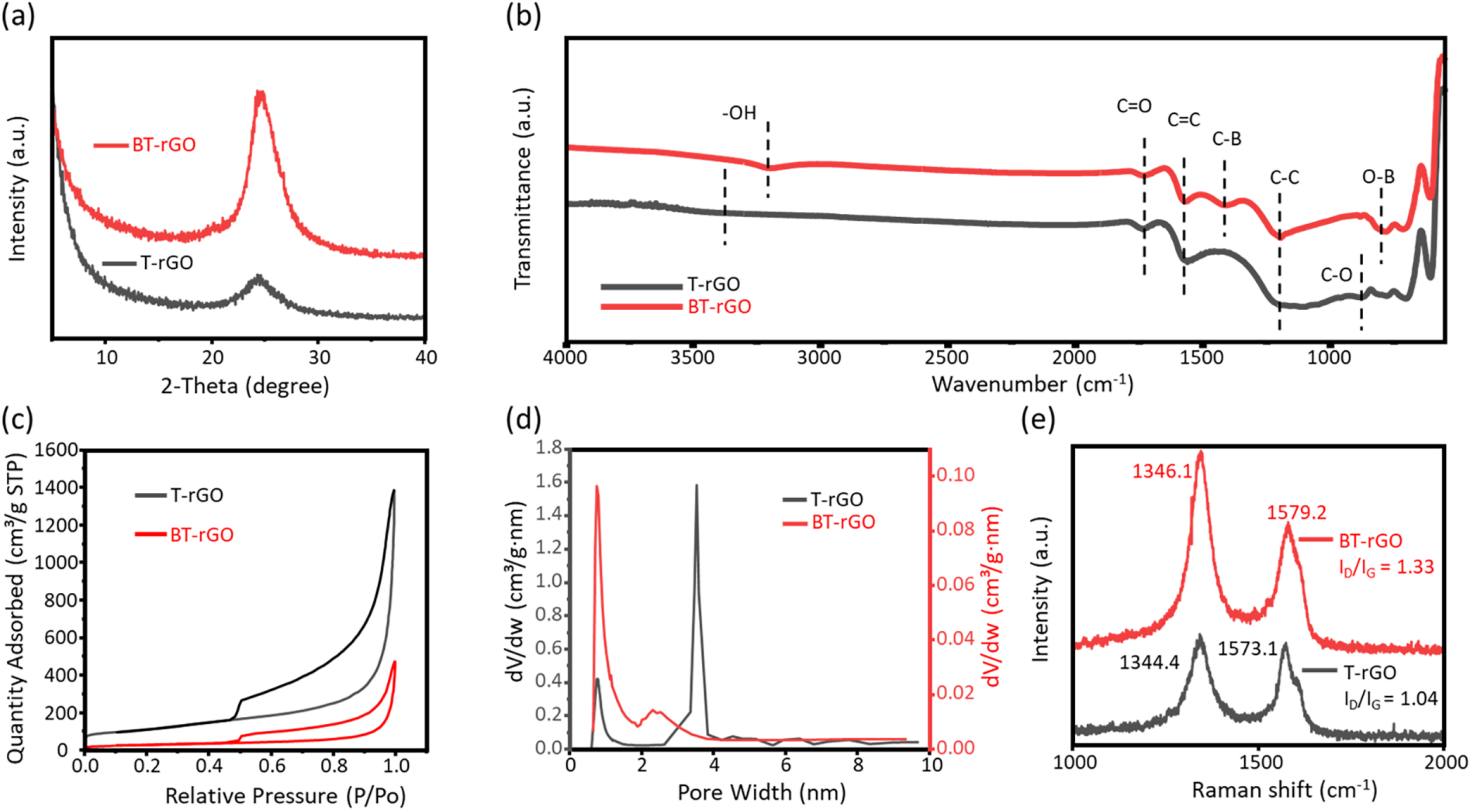
(a) XRD pattern, (b) FT-IR spectra, (c) nitrogen adsorption–desorption isotherms, (d) pore size distribution, and (e) Raman spectra of T-rGO and BT-rGO powders.

Boron-doped reduced graphene oxide (BT-rGO) and graphene oxide suspension (GO) was integrated via a redox reaction using a Zn plate. Figure S4 shows that the pristine Zn plate exhibits a relatively smooth surface with characteristic metallic polishing lines. After the reaction, the surface becomes significantly roughened, displaying layered and wrinkled structures, indicating the formation of an interfacial reaction layer. In addition, Figure S5 reveals the emergence of a weak but distinguishable peak at approximately 437 cm^–1^, which corresponds to the E_2_ (high) vibrational mode of hexagonal ZnO, and 581.2 cm^–1^ (E_1_ mode) after the reaction. As a result, the combined SEM and Raman results provide clear evidence that surface oxidation of the Zn plate occurs during the reaction process. Figure 2a–c show SEM images of BT-rGO_3_/Zn-rGO_1_ hydrogel buckypaper, revealing prominent surface wrinkles and porous structures compare with BT-rGO_1_/Zn-rGO_1_ and BT-rGO_2_/Zn-rGO_1_ as shown in Figure S6, demonstrating partial layer delamination and micro voids creation. Figures 2d and S7 show that EDS mapping confirms the homogeneous distribution of boron, demonstrating that the GO solution successfully dispersed BT-rGO during hydrogel buckypaper formation. However, BT-rGO_4_/Zn-rGO_1_ failed to form a free-standing hydrogel buckypaper, as shown in Figure S2b, indicating that the proportion of BT-rGO plays a critical role in hydrogel buckypaper formation. With an increasing boron content, significant structural evolution is also observed in XRD and Raman analyses. In Figure 2e, the XRD (002) reflection peaks for BT-rGO_1_/Zn-rGO_1_, BT-rGO_2_/Zn-rGO_1_, and BT-rGO_3_/Zn-rGO_1_ appear at 25.0°, 24.8°, and 24.5°, respectively, systematically shifting toward lower angles, indicating expansion of the interlayer spacing. The broader peak width further signifies a disrupted stacking order and increased turbostratic disorder.^52^ Meanwhile, the Raman spectra in Figure 2f show a progressive increase in the I_D_/I_G_ ratio, which are 1.46, 1.47, and 1.49 of BT-rGO_1_/Zn-rGO_1_, BT-rGO_2_/Zn-rGO_1_, and BT-rGO_3_/Zn-rGO_1_, respectively, confirming higher defect density and greater disruption of sp^2^ conjugation.^53,54^ The corrugated and defect-rich BT-rGO sheets disrupt interlayer restacking. As the BT-rGO fraction increases, the collective phonon modes of the stacked network become progressively softer, leading to a net redshift of both the D and G bands.

**Figure 2:**
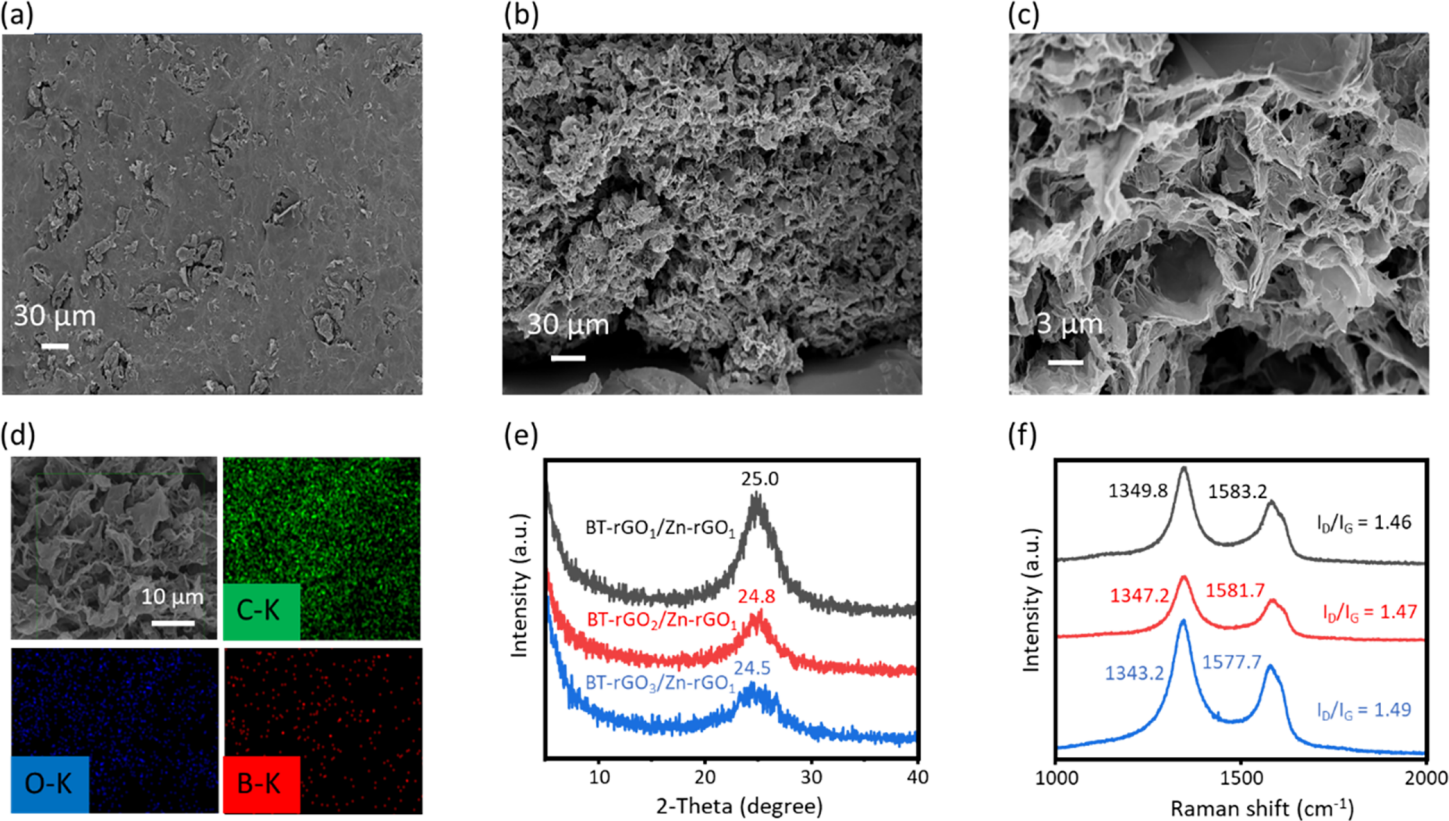
(a) Top-view of SEM image, (b) side-view of SEM image, (c) zoom in SEM image of (b), and (d) elemental mapping of BT-rGO_3_/Zn-rGO_1_ hydrogel buckypaper; (e) XRD pattern, and (f) Raman spectra of BT-rGO_1_/Zn-rGO_1_, BT-rGO_2_/Zn-rGO_1_, and BT-rGO_3_/Zn-rGO_1_ hydrogel buckypapers.

Having established that GO facilitates the formation of a continuous, defect-mitigated BT-rGO film, the energy-storage properties of the resulting hydrogel buckypapers were subsequently evaluated. Each sample was subjected to an activation process by applying constant potentials of −1.2 and 2.0 V for 180 s. By comparing the charge-storage performance before and after activation, the pristine Zn-rGO (Figure 3a), T-rGO/Zn-rGO (Figure 3b), and BT-rGO_1_/Zn-rGO_1_ (Figure 3c) buckypaper exhibit capacitance enhancements of 39.32%, 58.76%, and 63.48%, respectively. Upon further increasing the boron content in the hydrogel buckypaper, the BT-rGO_3_/Zn-rGO_1_ system reached an 86.09% enhancement, confirming that boron incorporation plays a dominant role in promoting charge-storage capability. To elucidate the role of hydrophilicity of the B-doped GO hydrogel buckypaper in enhancing the energy-storage performance, the moisture absorption behavior of BT-rGO_3_/Zn-rGO_1_ was investigated. Figure 3f shows that the weight of BT-rGO powder continuously increases with air-exposure time, indicating its high surface polarity. In addition, Figure S8 reveals that the −OH peak intensity of BT-rGO powder increases with prolonged air exposure, demonstrating that electron-deficient boron centers facilitate a nucleophilic attack by water molecules, resulting in the formation of additional −OH and C–O functional groups on the surface. This enhanced hydrophilicity provides a mechanistic explanation for the excellent electrolyte observed during the electrochemical activation process. In Figure S9a, the XRD (002) reflections of before and after electrochemical activation samples show a negligible shift in peak position around 24.5°, suggesting that the interlayer spacing and graphitic stacking remain essentially intact. This indicates that activation did not significantly alter long-range ordering within the carbon layers. Prior to the electrochemical activation process, the higher *I*_D_/*I*_G_ ratios in B-rich samples indicate a high density of defect-related active sites, including edge defects, vacancies, and B-induced lattice distortions. These sites act as electrochemically addressable reaction centers during the electrochemical activation process, enabling faster formation of oxygen functionalities and improve ionic pathways. The additional disorder introduced by boron also enhances ion storage sites by increasing local reactivity. In contrast, in Figure S9b, the Raman spectra underwent substantial modifications. The *I*_D_/*I*_G_ ratio decreases after activation (from 1.49 to 1.45), suggesting partial healing of lattice distortions and reorganization of the sp^2^ network. Meanwhile, both the D and G bands exhibit clear redshifts (the D band from 1343.2 to 1340.5 cm^–1^; the G band from 1577.7 to 1573.7 cm^–1^), which reflects relaxation of local strain and strengthening of extended π conjugation as oxygen functionalities are incorporated more uniformly. The redshift also implies that activated films possess more interconnected domains with reduced vibrational energy, consistent with improved structural continuity. Figure S10 shows contact angle measurements of E-BT-rGO_1_/Zn-rGO_1_, E-BT-rGO_2_/Zn-rGO_1_, and E-BT-rGO_3_/Zn-rGO_1_ electrodes. The measured contact angles are 74.1°, 67.2°, and 61.6°, respectively. E-BT-rGO_3_/Zn-rGO_1_ exhibits the smallest contact angle among the three samples, indicating the highest surface hydrophilicity. These results reveal that electrochemical activation induces a transformation from a defect-dominated structure to a partially reorganized and more interconnected carbon framework in boron-doped hydrogel buckypaper, owing to its higher density of reactive defect sites and enhanced surface polarity and wettability, thereby accounting for its superior activation efficiency and electrochemical performance.^55^

**Figure 3:**
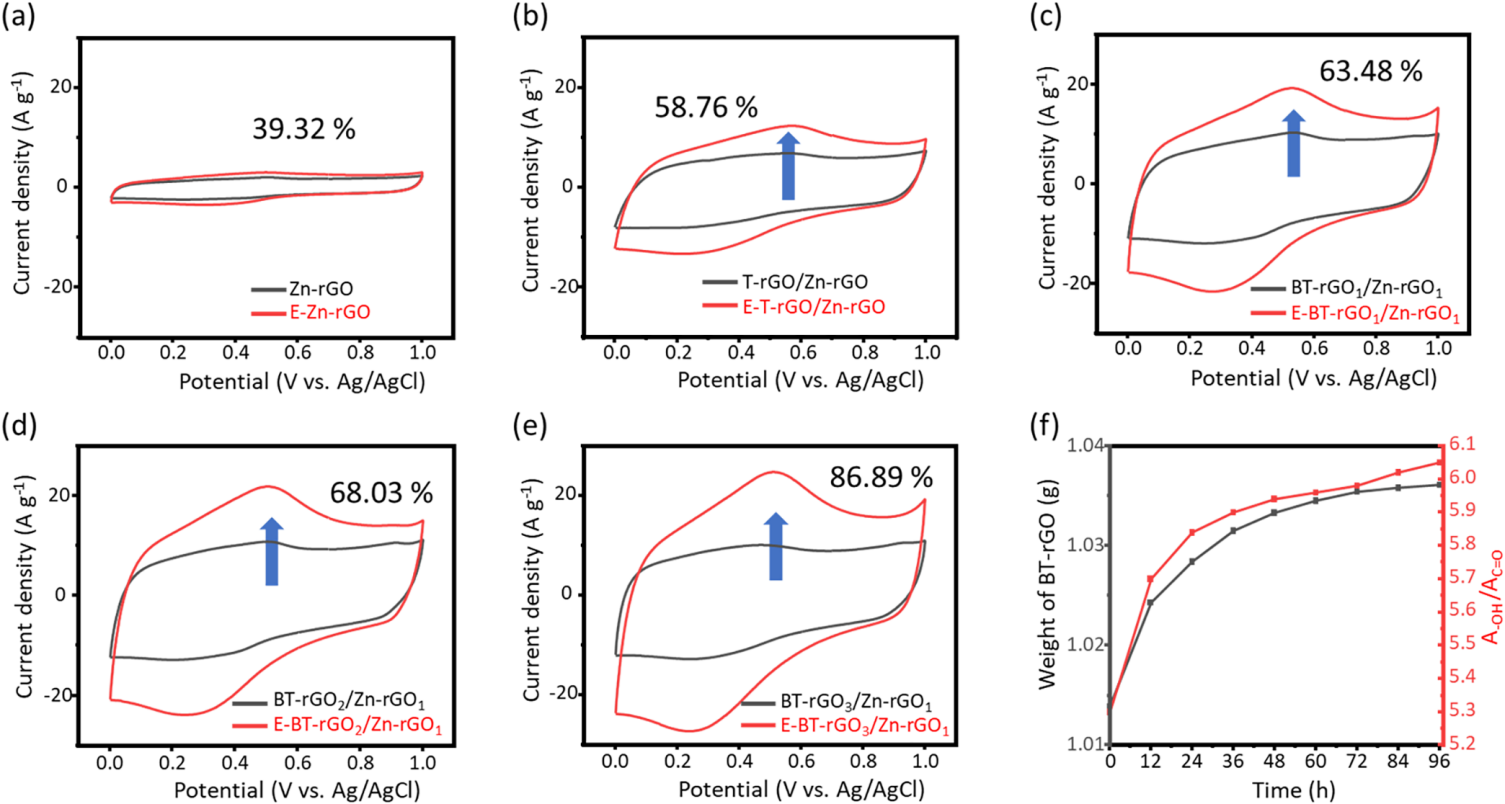
CV analysis and structural stability. CV curves at 50 mV s^–1^ of (a) Zn-rGO, (b) T-rGO/Zn-rGO, (c) BT-rGO_1_/Zn-rGO_1_, (d) BT-rGO_2_/Zn-rGO_1_, and (e) BT-rGO_3_/Zn-rGO_1_ before (black) and after (red, labeled with “E-“) electrochemical enhancement. (f) The weight and −OH peak transmittance in FT-IR variation of BT-rGO powder stay in air at different times.

To understand the chemical origins of the defect types and oxygen incorporation, XPS and FT-IR analyses provide evidence of how activation modulates surface functionalities at boron-adjacent carbon sites. In Figure 4a, for the BT-rGO_3_/Zn-rGO_1_ hydrogel buckypaper, XPS analysis before and after electrochemical enhancement provides a deep insight into the chemical modification occurring on the carbon surface. The overall oxygen content increases significantly from 15.3% to 19.2%, indicating that the activation process drives the incorporation of additional oxygen functionalities. This increase reflects the electrochemical oxidation of defect sites, edge carbon atoms, and boron-adjacent regions that are highly reactive due to electron deficiency introduced by boron doping. In Figure 4b, the O=C–O, C=O, C–O, C=C, and C–B bonds locate at 288.6, 286.2, 285.2, 284.8, and 284.2 eV in the C 1s spectra, respectively, which provide more detailed evidence of this transformation. Before activation, the C–O/C–B and C=O/C–B intensity ratios are relatively low (3.12 and 2.10), consistent with a partially reduced rGO framework. After enhancement, both ratios increase dramatically to 5.44 and 4.78, respectively, demonstrating the formation of new C–O (epoxy/hydroxyl) and C=O (carbonyl) groups. The clear growth of these peaks suggests that specific carbon sites adjacent to B–C or B–O motifs are preferentially oxidized, likely due to the local polarization effect created by electron-deficient boron atoms, which enhances the susceptibility of neighboring carbon atoms to nucleophilic attack or electrochemical oxidation. Similarly, in Figure 4c, the B–O, C=O, and C–O bonds locate at 534.0 533.1, and 531.8 eV, respectively, in the spectral data of the O 1s spectra, which exhibits parallel trends. The O=C/O–B and C–O/B–O ratios increase from 1.63 and 2.12 to 3.38 and 3.18 following electrochemical activation. This change confirms that more oxygen atoms are bonded directly to the carbon lattice rather than altering the B–O environment. The strengthened O=C signal indicates more carbonyl-type structures, while the increase in the C–O component reflects growth of hydroxyl and epoxy functionalities. These trends collectively reveal that the carbon framework undergoes substantial chemical functionalization, driven by the electrochemical enhancement process. In contrast, Figure 4d shows that the B 1s spectra of BC_3_, BC_2_O, and BCO_2_ species appear at 190.1, 192.5, and 195.0 eV, respectively, with minimal changes in their relative contributions before and after electrochemical enhancement, indicating that these B–C and B–O species remain chemically anchored within the carbon matrix and primarily act as stable coordination centers that facilitate oxygen incorporation on neighboring carbon atoms. This observation supports the view that boron acts as an electronic modulator rather than a reactive species, promoting oxygen uptake after electrochemical enhancement. The XPS spectra and high-resolution spectra of C 1s, O 1s, and B 1s of BT-rGO_1_/Zn-rGO_1_ and BT-rGO_2_/Zn-rGO_1_ hydrogel buckypapers are shown in Figure S11, and elemental contents are demonstrated in Table S2. In Figure 4e, complementary FT-IR analysis further corroborates these findings. The absorption bands associated with B–C (1068 cm^–1^) and B–O (880 cm^–1^) bonds were clearly present in both spectra, confirming the structural stability of B-related bonding motifs. After enhancement, the −OH stretching band (3350 cm^–1^) and the C–O vibration (1201 cm^–1^) intensify markedly, consistent with the XPS-detected increase in oxygen functionalities. The strengthened −OH signal indicates enhanced surface hydrophilicity, while the enrichment of C–O and C=O groups suggests that the electrochemical process introduced new polar functionalities across the carbon network. These results collectively demonstrate that enhancement leads to substantial oxygen incorporation, while boron serves as an unaltered but crucial facilitator of this transformation. Taken together, these results demonstrate that electrochemical enhancement primarily oxidizes defect-rich carbon sites, while preserving the structural integrity of the boron coordination environment. Instead of undergoing structural transformation, boron functions as a stable electronic modulator that promotes selective oxygen incorporation, thereby supporting the chemical functionalization inferred from the observed activation behavior.

**Figure 4:**
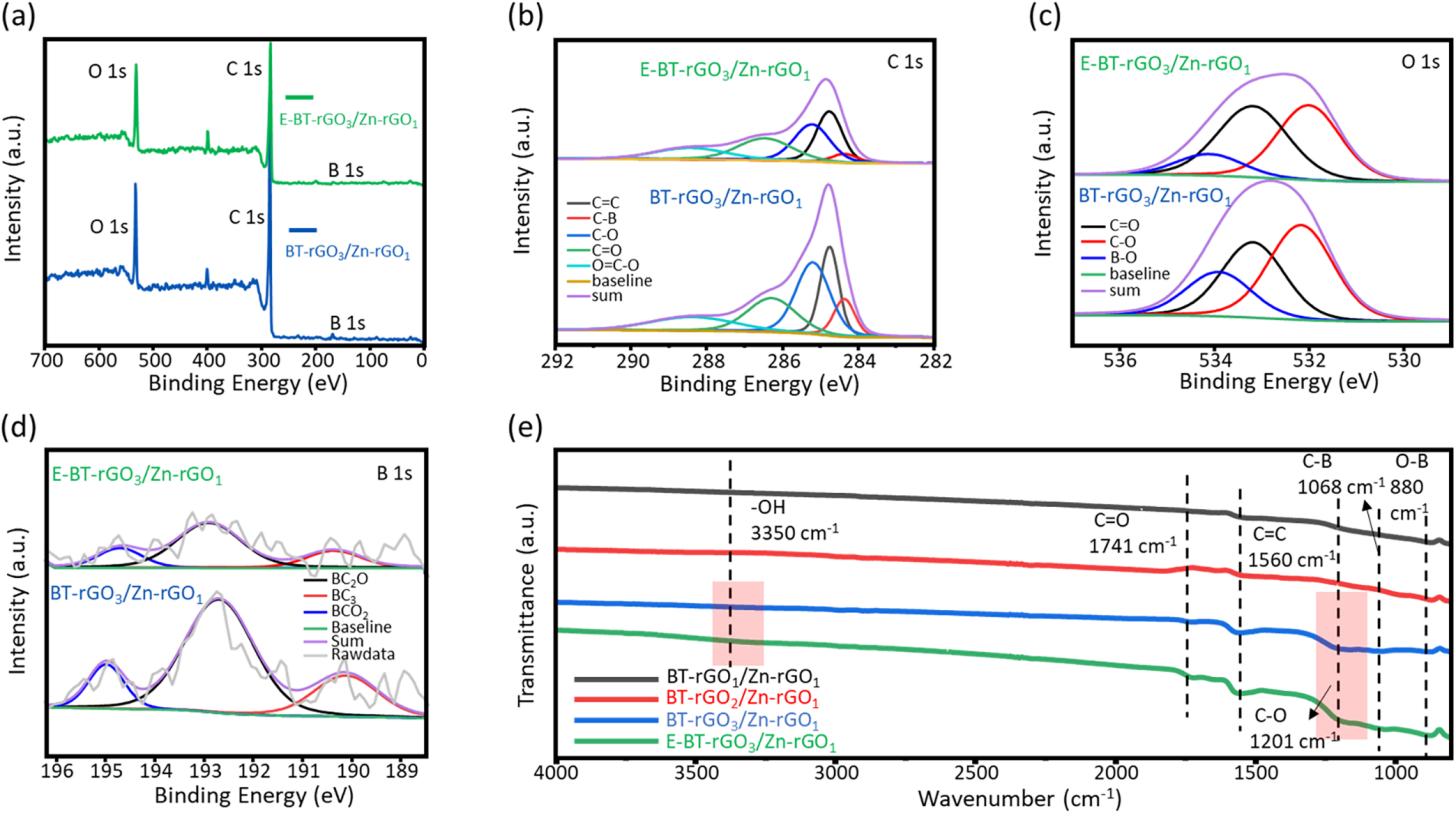
(a) XPS spectra, and high-resolution spectra of (b) C 1s, (c) O 1s, and (d) B 1s of BT-rGO_3_/Zn-rGO_1_ and E-BT-rGO_3_/Zn-rGO_1_; (e) FT-IR spectra of BT-rGO_1_/Zn-rGO_1_, BT-rGO_2_/Zn-rGO_1_, BT-rGO_3_/Zn-rGO_1_, and E-BT-rGO_3_/Zn-rGO_1_.

After elucidating the activation mechanism, comprehensive electrochemical evaluations are performed to examine how boron doping and activation jointly influence charge-storage behavior. As shown in Figure 5a,b, at a scan rate of 50 mV s^–1^ and a current density of 2 mA cm^–2^, E-BT-rGO_1_/Zn-rGO_1_, E-BT-rGO_2_/Zn-rGO_1_, and E-BT-rGO_3_/Zn-rGO_1_ electrodes exhibit quasi-rectangular CV profiles and nearly triangular GCD curves, which are indicative of surface-controlled pseudocapacitive behavior, and their electrochemical performances are dependent on their own loading mass shown in Table S3. The well-preserved rectangularity implies rapid ion adsorption–desorption kinetics and minimal internal diffusion resistance. Among the three samples, the E-BT-rGO_3_/Zn-rGO_1_ electrode displays the highest specific capacitance and the longest discharge time. In Figure 5c,d, the E-BT-rGO_3_/Zn-rGO_1_ electrode maintains well-defined CV curves from 1–100 mV s^–1^ without obvious distortion, indicating that the capacitive response is retained even under extremely fast polarization. Likewise, stable and symmetric GCD profiles are maintained from 2–100 mA cm^–2^, demonstrating that the electrode preserves efficient charge transfer and ionic accessibility across a broad range of operating conditions. In Figure S12, the E-BT-rGO_1_/Zn-rGO_1_ and E-BT-rGO_2_/Zn-rGO_1_ electrodes show the same observation at different scan rates and current densities in CV and GCD test. In Figure 5e, at a current density of 2 mA cm^–2^, the E-BT-rGO_3_/Zn-rGO_1_ electrode achieves a maximum specific capacitance of 443.63 F g^–1^, significantly higher than E-BT-rGO_1_/Zn-rGO_1_ (323.31 F g^–1^) and E-BT-rGO_2_/Zn-rGO_1_ (372.46 F g^–1^). Even at a current density of 100 mA cm^–2^, the E-BT-rGO_3_/Zn-rGO_1_ electrode retains a specific capacitance of 309.38 F g^–1^, still higher than E-BT-rGO_1_/Zn-rGO_1_ (250.0 F g^–1^) and E-BT-rGO_2_/Zn-rGO_1_ (238.46 F g^–1^). The E-BT-rGO_3_/Zn-rGO_1_ electrode retains 69.74% of its initial capacitance, exceeding the retention values of E-BT-rGO_1_/Zn-rGO_1_ (66.74%) and E-BT-rGO_2_/Zn-rGO_1_ (67.12%). Table S4 compares the specific capacitance of heteroatom-doped rGO and related carbon-based materials, highlighting that the simultaneously formed, binder-free, free-standing E-BT-rGO_3_/Zn-rGO_1_ hydrogel buckypaper exhibits outstanding specific capacitance. In Figure 5f, the Nyquist plot reveals that E-BT-rGO_3_/Zn-rGO_1_ possesses the lowest equivalent series resistance (*R*_s_ = 0.45 Ω) and charge-transfer resistance (*R*_ct_ = 0.22 Ω), values substantially below those of E-BT-rGO_2_/Zn-rGO_1_ (*R*_s_ = 0.66 Ω and *R*_ct_ = 0.45 Ω) and E-BT-rGO_1_/Zn-rGO_1_ (*R*_s_ = 0.70 Ω and *R*_ct_ = 0.56 Ω). The decrease in *R*_s_ indicates improved electrode–electrolyte interfacial resistance and enhanced intrinsic conductivity owing to the reconstruction of sp^2^ domains during activation. The reduced R_ct_ confirms that boron-induced polarity leads to faster interfacial redox reactions. Finally, in Figure 5g, the long-term cycling stability test conducted at 50 mA cm^–2^ shows that the E-BT-rGO_3_/Zn-rGO_1_ electrode has 88% of capacitance retention after 10,000 cycles. Figure S13 shows that the (002) diffraction peak of E-BT-rGO_3_/Zn-rGO_1_ remains unchanged, and Figure S14 presents the SEM images revealing that the porous network structure remains intact without observable collapse, cracking, or delamination before and after stability testing, demonstrating exceptional structural stability and effective suppression of carbon framework degradation. Moreover, Figure S15 shows that the Raman spectra show no significant variation in the D/G intensity ratio (*I*_D_/*I*_G_ = 1.45) and peak positions, indicating that the defect density and graphitic structure are well preserved after prolonged cycling. The stabilized B–C and B–O bonding motifs, as confirmed by XPS and FT-IR, act as anchoring sites that maintain the integrity of the carbon lattice during repeated ion insertion/extraction.

**Figure 5:**
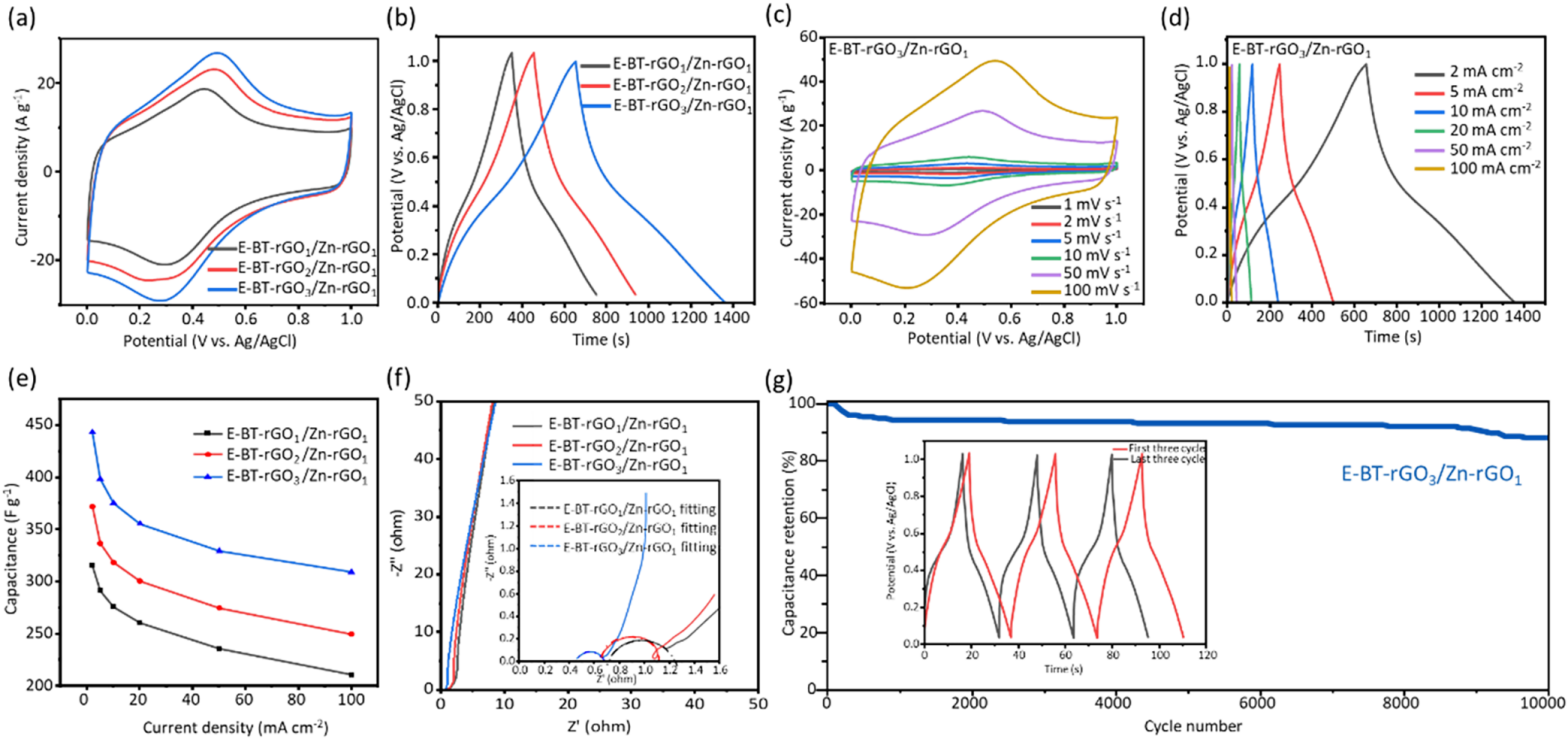
(a) The CV curves at 50 mV s^–1^, and (b) GCD curves at 2 mA cm^–2^ of E-BT-rGO_1_/Zn-rGO_1_, E-BT-rGO_2_/Zn-rGO_1_, and E-BT-rGO_3_/Zn-rGO_1_; (c) the CV curves at 1–100 mV s^–1^, and (d) GCD curves at 2–100 mA cm^–2^ of E-BT-rGO_3_/Zn-rGO_1_; (e) the specific capacitance of E-BT-rGO_1_/Zn-rGO_1_, E-BT-rGO_2_/Zn-rGO_1_, and E-BT-rGO_3_/Zn-rGO_1_ at 2–100 mA cm^–2^; (f) Nyquist plot of E-BT-rGO_1_/Zn-rGO_1_, E-BT-rGO_2_/Zn-rGO_1_, and E-BT-rGO_3_/Zn-rGO_1_; and (g) the cycling test at 50 mA cm^–2^ over 10,000 cycles of the E-BT-rGO_3_/Zn-rGO_1_ electrode.

To further clarify the charge-storage kinetics, a detailed kinetic analysis is performed by using the power-law relationship between the peak current (*i*) and the scan rate (v) in cyclic voltammetry. This relationship follows the equation^56,57^

$$i=av^{b}\left(0.5\leq b\leq 1\right)$$

where the exponent *b* provides direct insight into the dominant charge-storage mechanism. A value of *b* = 1 corresponds to a surface-controlled capacitive process, indicating that current responses scale linearly with scan rate due to rapid ion adsorption/desorption on accessible active sites. Conversely, *b* = 0.5 represents a diffusion-controlled process dominated by slow ion intercalation into the inner pores or bulk regions. As shown in Figures 6a–6c, the log­(*i*) versus log­(v) plots of E-BT-rGO_1_/Zn-rGO_1_, E-BT-rGO_2_/Zn-rGO_1_, and E-BT-rGO_3_/Zn-rGO_1_ hydrogel buckypaper exhibit excellent linearity across the tested scan rates, confirming reliable kinetic fitting. The extracted oxidation b values of 0.91, 0.91, and 0.92, respectively, indicate that all three electrodes charge-storage behavior predominantly through surface-controlled processes. The slightly higher b value for the E-BT-rGO_3_/Zn-rGO_1_ electrode suggests that activation and increased boron incorporation generate a more accessible electroactive surface, consistent with its enlarged Raman *I*_D_/*I*_G_ ratio (Figure 2f) and enhanced hydrophilicity shown in FT-IR and XPS analyses (Figure 4). These features enhance ion transport and reduce kinetic limitations, enabling charge storage that remains close to ideal pseudocapacitive behavior.

**Figure 6:**
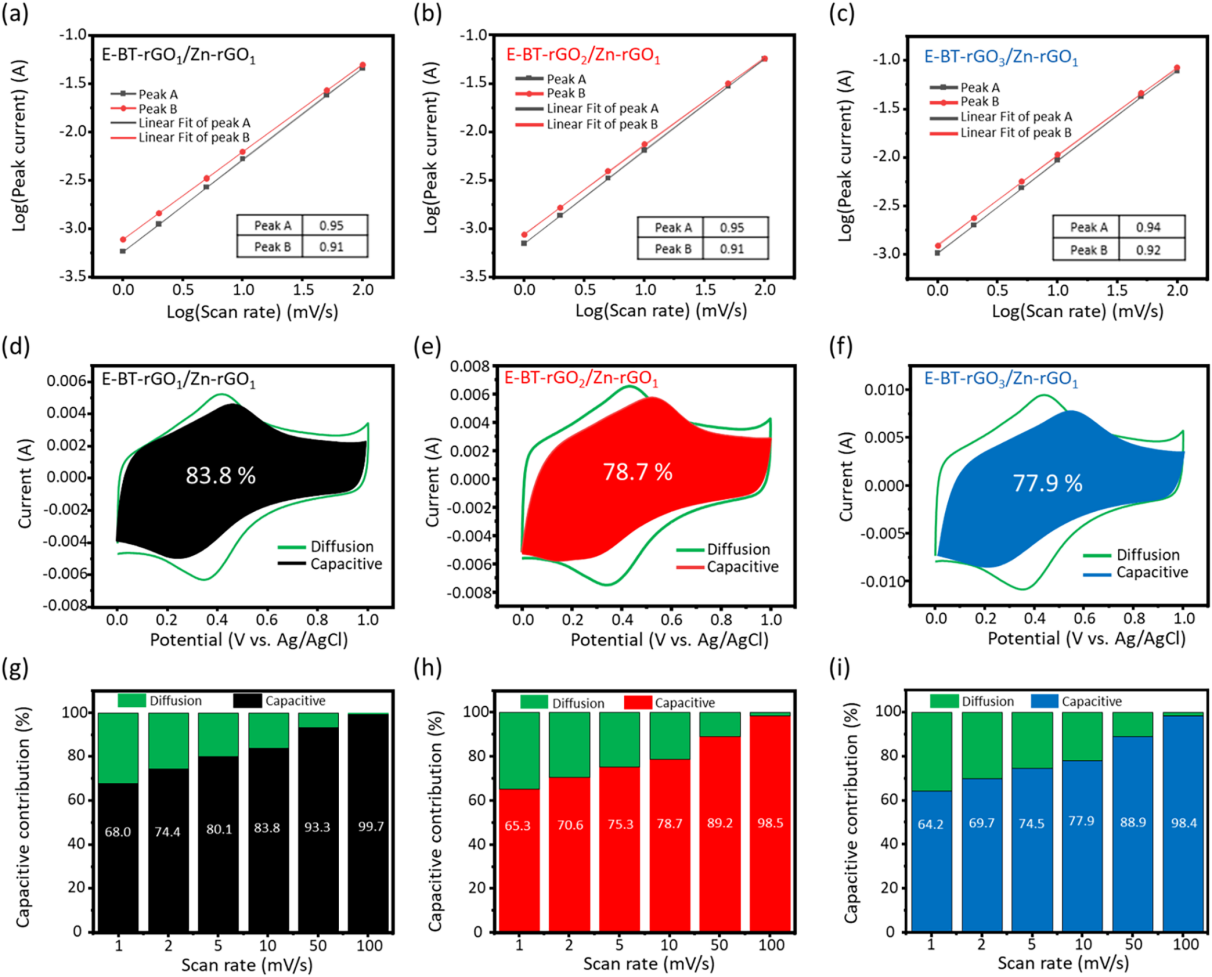
Dependence of oxidation (Peak B) and reduction (Peak A) b value for (a) E-BT-rGO_1_/Zn-rGO_1_, (b) E-BT-rGO_2_/Zn-rGO_1_, and (c) E-BT-rGO_3_/Zn-rGO_1_; The separation of capacitive and diffusion contributions for (d) E-BT-rGO_1_/Zn-rGO_1_, (e) E-BT-rGO_2_/Zn-rGO_1_, and (f) E-BT-rGO_3_/Zn-rGO_1_ at 10 mV s^–1^; Capacitance contribution of (g) E-BT-rGO_1_/Zn-rGO_1_, (h) E-BT-rGO_2_/Zn-rGO_1_, and (i) E-BT-rGO_3_/Zn-rGO_1_ at different scan rates.

To further separate the capacitive and diffusion contributions, Dunn’s method was applied. The current responses at a given potential are expressed as^58,59^

$$i\left(V\right)=k_{1}v+k_{2}v^{1/2}$$

where *k*_1_v represents the capacitive current and *k*_2_v^1/2^ corresponds to the diffusion-controlled contribution. The method allows the quantification of how much charge originates from rapid surface processes (*k*_1_v) versus slower ion-diffusion processes (*k*_2_v^1/2^). As shown in Figure 6d–6f, at a moderate scan rate of 10 mV s^–1^, the capacitive contributions for E-BT-rGO_1_/Zn-rGO_1_, E-BT-rGO_2_/Zn-rGO_1_, and E-BT-rGO_3_/Zn-rGO_1_ hydrogel buckypaper are 83.8%, 78.7%, and 77.9%, respectively. The capacitive contributions of other hydrogel buckypaper scan rates are shown in Figures S16–S18. This trend indicates a gradual increase in diffusion-controlled charge storage as the proportion of BT-rGO increases. This behavior directly aligns with structural characterizations: Raman analysis (Figure 2f) shows that a higher boron content produced more disorder-related defects, which act as diffusion pathways or ion-hosting sites. Moreover, EIS measurement (Figure 5f) reveals that the BT-rGO_3_/Zn-rGO_1_ electrode exhibits the lowest *R*_s_ and *R*_ct_ values, signifying reduced resistance and faster ion transfer. Overall, these results confirm that boron incorporation facilitates deeper ion penetration into the electrode microstructure, enhancing the diffusion component in the overall capacitance. Additionally, in Figure 6g–6i, at higher scan rates, the capacitive contribution increases because the rapid potential changes favor surface redox reactions rather than ion diffusion into deeper pores.^60,61^ This behavior reflects the fast surface kinetics enabled by the activation-induced oxygen functionalities and boron-enhanced polarity of the carbon framework. Overall, the combined kinetic analyses demonstrate that increasing the boron content not only improves surface PC but also promotes ion diffusion, yielding a balanced and highly efficient charge-storage mechanism. These kinetic insights, when integrated with the structural and chemical evolution described earlier, establish that boron-mediated polarity and activation-induced oxygenation cooperatively regulate both surface and diffusion processes, thereby explaining the superior rate performance and capacitance of the E-BT-rGO hydrogel buckypapers.

Following the outstanding electrochemical performance of the E-BT-rGO_3_/Zn-rGO_1_ hydrogel buckypaper in the three-electrode configuration, its energy-storage behavior is further evaluated under a two-electrode system. As shown in Figure S19, the device maintains well-defined CV curves across scan rates from 1 to 100 mV s^–1^ and stable GCD profiles at current densities ranging from 2 mA to 100 mA cm^–2^. The nearly rectangular CV shapes and linear charge–discharge curves indicate that the electrode preserves efficient ion transport and rapid redox kinetics even when operated as a practical two-electrode cell. To construct a flexible all-solid-state supercapacitor, PVA-H_2_SO_4_ is used as a polymer electrolyte membrane (Figure S20). As shown in Figure 7a,b, the assembled solid-state device exhibits quasi-rectangular CV curves and symmetric GCD profiles, demonstrating that the flexible configuration supports fast ionic migration through the PVA gel matrix while maintaining stable electron conduction through the E-BT-rGO_3_/Zn-rGO_1_ electrode network. The absence of distortion in either curve further confirms that the device experiences minimal internal resistance and that the electrode/electrolyte interfaces remain well integrated after assembly. In Figure 7c, the tunability of the device is examined by connecting three identical flexible supercapacitors in parallel and in series. In the GCD curves at 10 mA cm^–2^, the parallel connection results in an approximately three times increase in discharge time, consistent with the additive behavior expected when capacitances are combined. Conversely, the series configuration successfully expands the operating voltage window by nearly three times, demonstrating that the devices can be modularly integrated to meet different power or voltage requirements. Similar trends are observed in the CV curves measured at 50 mV s^–1^ (Figure S21a), demonstrating the excellent scalability and electrical adaptability of the solid-state configuration. The mechanical robustness of the flexible device is investigated by bending it to various angles (0°, 30°, 60°, 90°, 120°, and 180°), as depicted in Figure 7d. The device maintains structural integrity without delamination or cracking under deformation. Correspondingly, the GCD curves at 10 mA cm^–2^ (Figure 7e) exhibits almost identical profiles at all bending angles, and the CV curves at 50 mV s^–1^ (Figure S21b) also remains identical. Additionally, Figure S22 shows repeated 1,200 bending cycles at large deformation angles; the device retains about 86.0% of its initial capacitance, indicating that the electrode maintained continuous electron pathways and stable electrolyte penetration even under mechanical stress. The figure further confirms that the solid-state supercapacitor exhibits excellent flexibility and mechanical durability, which could be potentially suitable for wearable or foldable electronics applications. Figure S23 displays that the device exhibits a slightly increased specific capacitance at 55 °C compared with room temperature operation, indicating good thermal tolerance within the targeted operating range. Figure 7f shows the Ragone plot of the present device with previously reported heteroatom-doped carbon-based symmetric supercapacitors. Based on GCD-derived values, the device delivers a maximum energy density of 16.96 Wh kg^–1^ at a power density of 0.12 kW kg^–1^, and a maximum power density of 5.21 kW kg^–1^ at an energy density of 6.77 Wh kg^–1^. These values surpass those of many heteroatom-doped carbon-based composite materials (PB-rGO,^62^ A-BNQD/rGO,^63^ N,B-rGO,^64^ PN-rGO,^65^ AMP–B-Fe,^66^ B/N-CNS,^67^ N-GQDs,^68^ CRG^69^). Finally, to demonstrate practical applicability, two flexible solid-state devices are connected in series to power seven commercial LED bulbs simultaneously, as shown in Figure 7g. The LEDs light brightly and steadily, confirm that the device can deliver sufficient voltage and stable output for real-world electronic applications.

**Figure 7:**
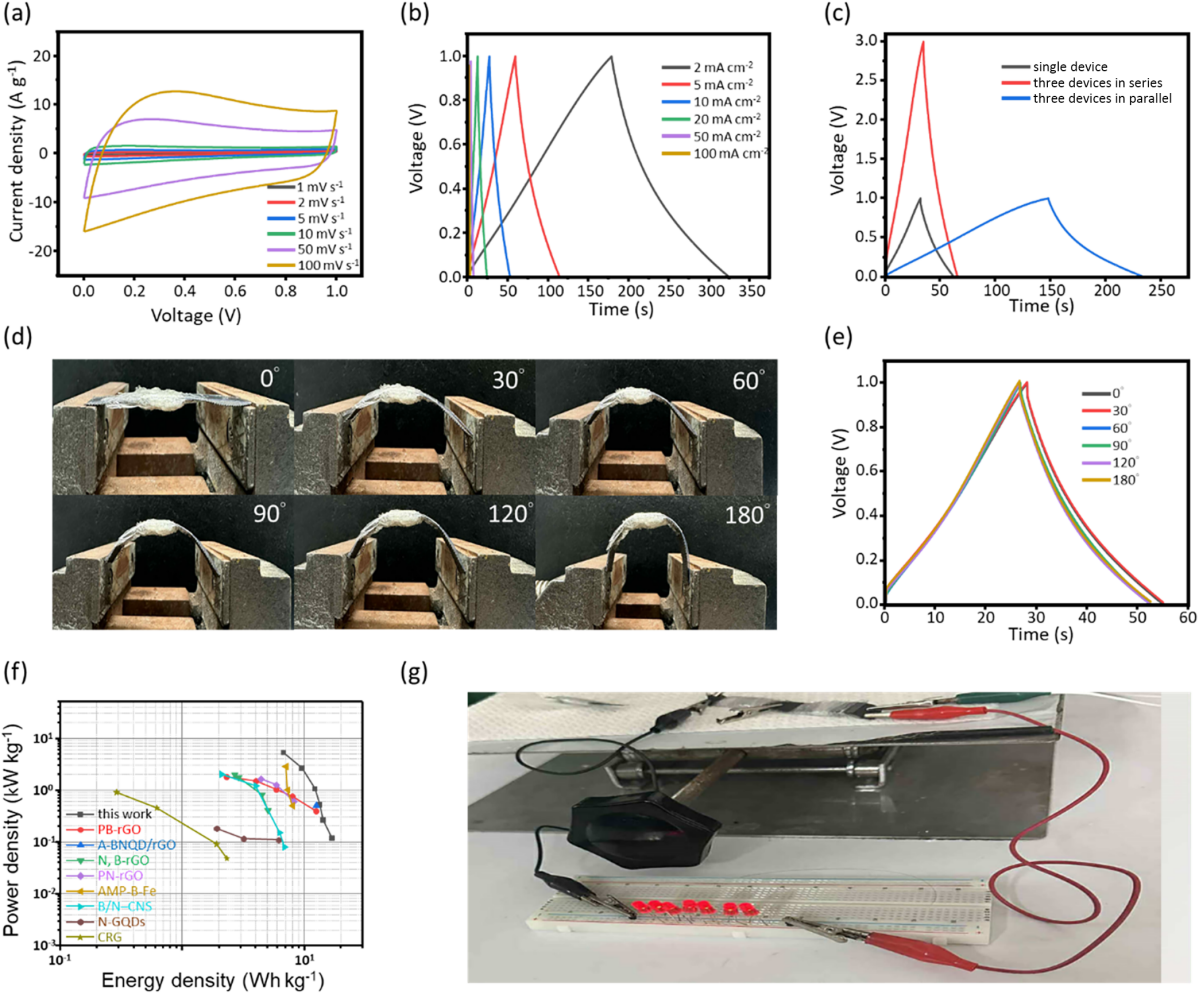
Flexibility of the solid-state supercapacitor based on E-BT-rGO_3_/Zn-rGO_1_ hydrogel buckypapers: (a) CV curves at 1–100 mV s^–1^, (b) GCD curves at 2–100 mA cm^–2^, (c) GCD curves of three series-connected and three parallel-connected devices at 10 mA cm^–2^, (d) photos of devices bent under 0°, 30°, 60°, 90°, 120°, and 180°, (e) GCD curves at different bending angles at 10 mA cm^–2^, (f) Ragone plot comparing the different carbon materials which previously reported, and (g) red LED powered by two supercapacitors in series.

## 4 Conclusion

In this work, we successfully fabricated a binder-free, free-standing carbon-based E-BT-rGO/Zn-rGO hydrogel buckypaper through a spontaneous metal-plate redox reaction followed by electrochemical activation. Boron incorporation plays a pivotal role in regulating both structure and chemistry: it prevents the severe restacking typically observed in graphene-based materials while simultaneously introducing abundant electrochemically active sites. In the three-electrode configuration, the optimized E-BT-rGO_3_/Zn-rGO_1_ composite electrode delivers a high specific capacitance of 443.63 F g^–1^ at 2 mA cm^–2^, along with excellent cycling stability—preserving 88% of its capacitance after 10,000 cycles at 50 mA cm^–2^. When assembled into a flexible solid-state supercapacitor using a PVA-H_2_SO_4_ gel electrolyte, the E-BT-rGO_3_/Zn-rGO_1_ device achieves a maximum energy density of 16.96 Wh kg^–1^ and a maximum power density of 5.21 kW kg^–1^, outperforming many previously reported heteroatom-doped carbon-based systems. Furthermore, the device maintains its electrochemical characteristics under a wide range of bending angles, demonstrating excellent mechanical flexibility. Its practical applicability is further verified by successfully powering a series of LED bulbs when two devices are connected in series. Overall, this study highlights the crucial role of boron doping in promoting electrochemical activation, enhancing interfacial charge transfer and improving ion transport within graphene frameworks. Furthermore, we demonstrate that coupling boron engineering with a metal-plate-driven redox strategy offers a simple yet effective pathway for producing binder-free, free-standing hydrogel buckypapers. These findings establish a promising and scalable approach to the development of next-generation flexible, high-performance supercapacitors.

## Supplementary Material



## Notes

XPS, SEM, FT-IR, XRD, and Raman analyses of B-doped GO and electrochemical and stability analyses of B-doped GO at different electrolyte conditions (PDF)
