# Supplementary material for: Free-Standing Boron-Doped Graphene Hydrogel Buckypaper Fabricated via Zinc-Plate Reduction for High-Performance Supercapacitors
Source: ACS Appl Mater Interfaces. 2026 Mar 26;18(13):19860–72. doi: 10.1021/acsami.6c00158 (PMC13067235; doi:10.1021/acsami.6c00158)
Supplement: Supplementary file 1 [file am6c00158_si_001.pdf]

Supporting Information

Free-standing Boron-doped Graphene Hydrogel Buckypaper Fabricated via Zinc-Plate Reduction for High-Performance Supercapacitors

Jia-Yu Ji[a], Jeremiah Hao Ran Huang[a], Shih-Wen Tseng[c], Yi-Wen Chen[d], Jhao-Rong Huang [c], and I-Wen Peter Chen[a,b,*]

[a]Department of Chemistry, National Cheng Kung University, No. 1, University Road, East District, Tainan 701, Taiwan

[b]Interdisciplinary Research Center on Material and Medicinal Chemistry, National Cheng Kung University, No. 1, University Road, East District, Tainan 701, Taiwan

[c]Core Facility Center, National Cheng Kung University, Tainan 701, Taiwan

[d]Graduate Institute of Biomedical Sciences, China Medical University, Taichung, Taiwan

* Corresponding author email: iwchen1978@gs.ncku.edu.tw

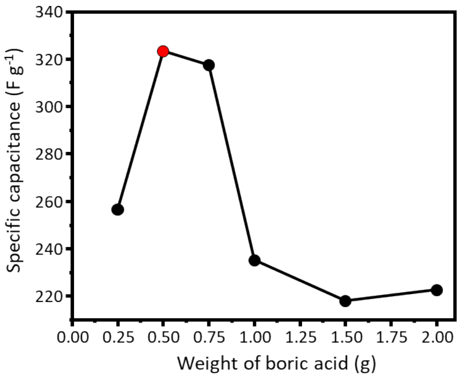

Figure S1. The specific capacitance of E-BT-rGO$_1$/Zn-rGO$_1$ film made by different weight of boric acid.

(a) (b)

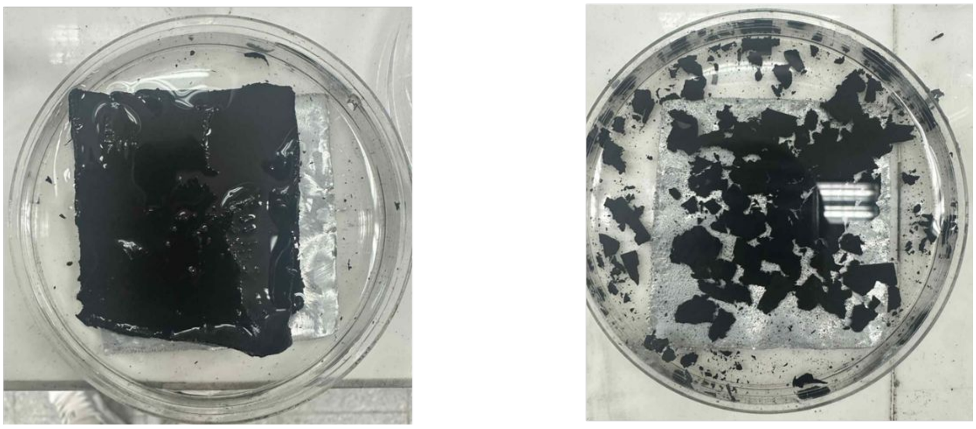

Figure S2. Photos of preparation the (a) BT-rGO$_3$/Zn-rGO$_1$ and (b) BT-rGO$_4$/Zn-rGO$_1$composite films.

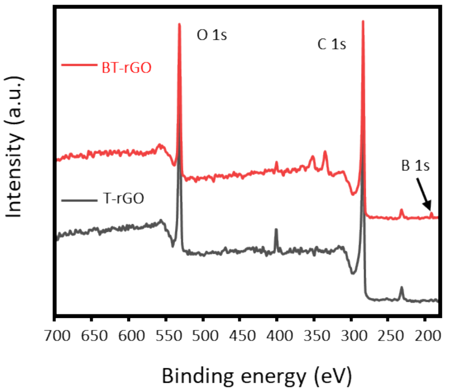

Figure S3. The XPS spectra of T-rGO and BT-rGO powder.

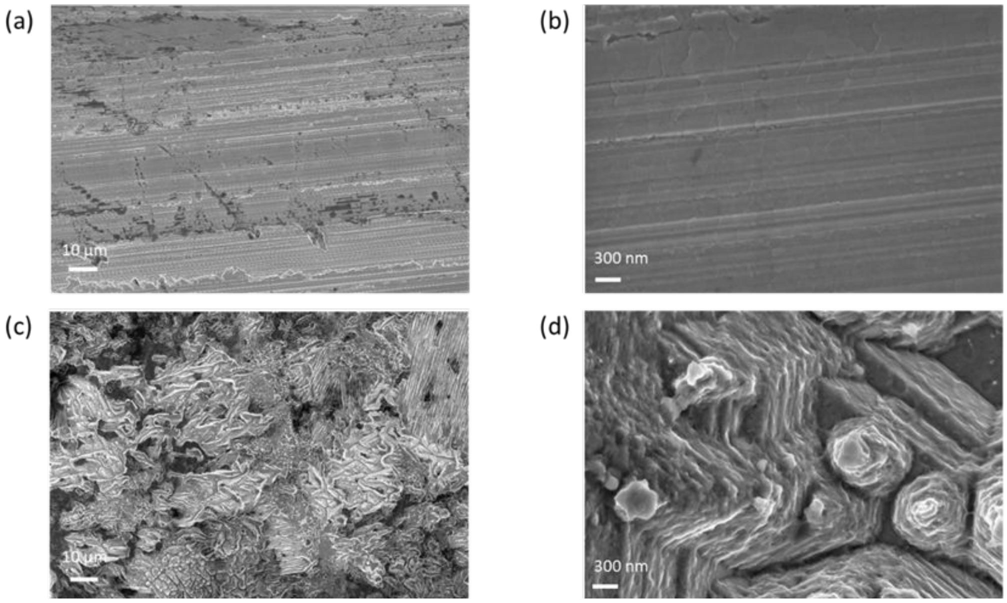

Figure S4. The SEM images of Zinc plate (a),(b) before the reaction and (c),(d) after the reaction.

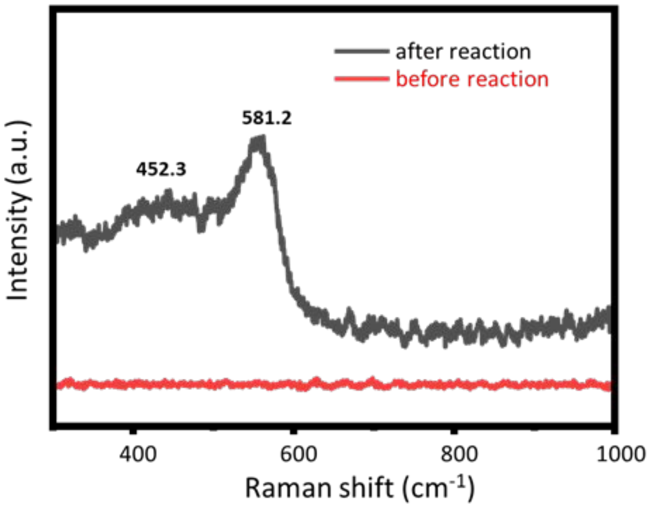

Figure S5. The Raman spectra of Zn plate before and after reaction.

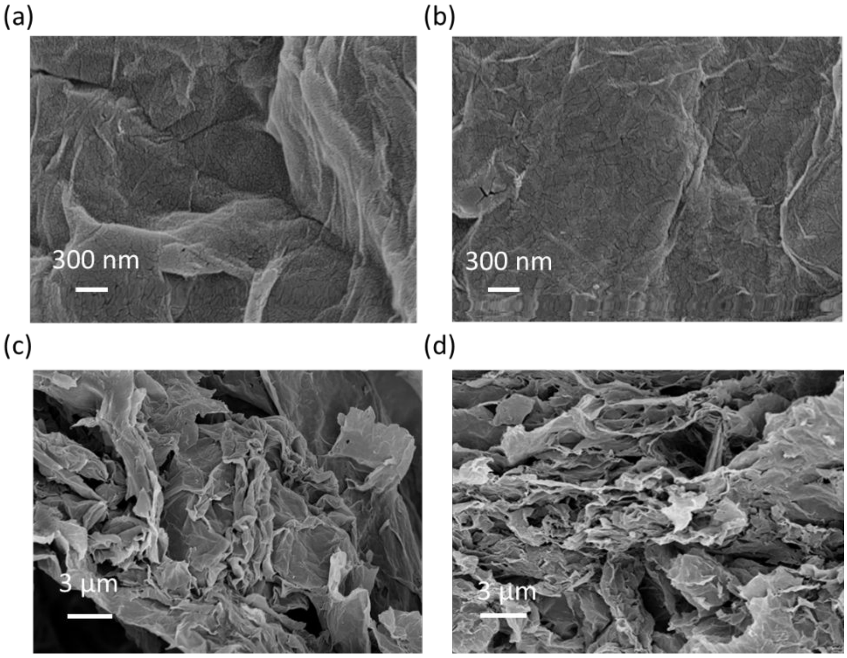

Figure S6. Top-view SEM image of (a) BT-rGO_1/Zn-rGO_1, and (b) BT-rGO_2/Zn-rGO_1; Side-view SEM image of (c) BT-rGO_1/Zn-rGO_1, and (d) BT-rGO_2/Zn-rGO_1.

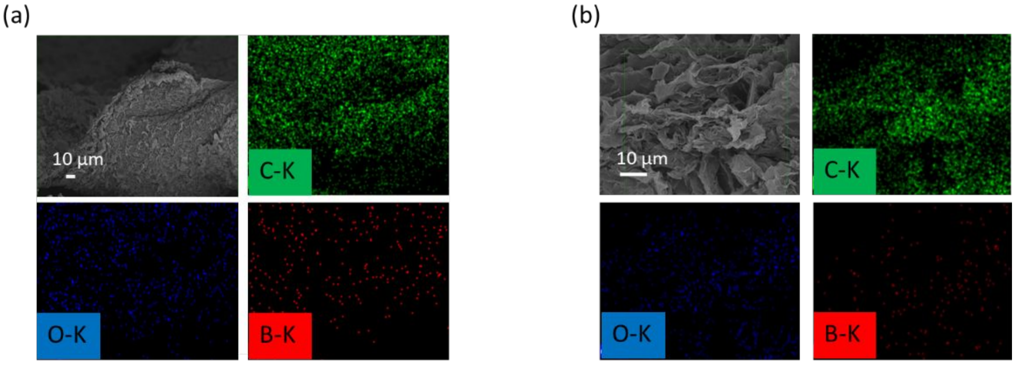

Figure S7. Elemental mapping of (a) BT-rGO_1/Zn-rGO_1, and (b) BT-rGO_2/Zn-rGO_1.

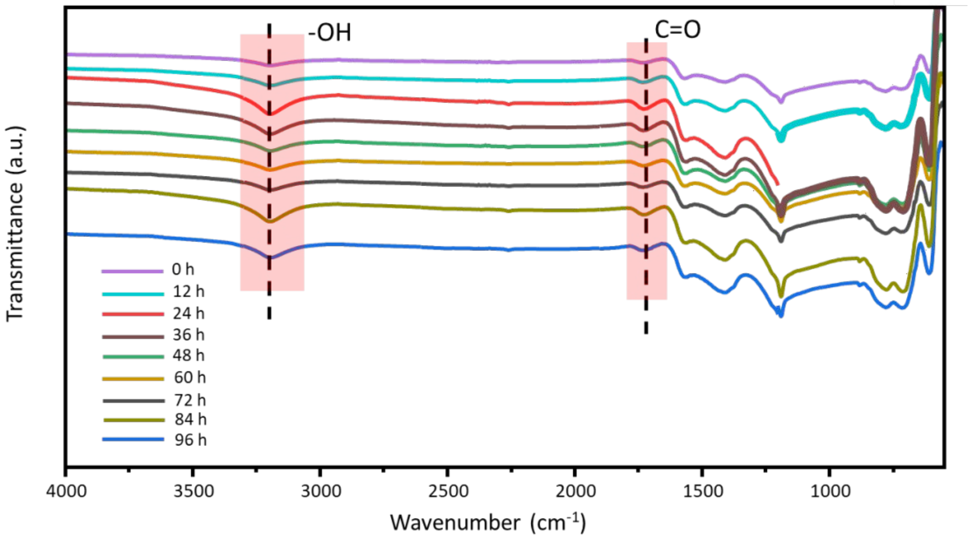

Figure S8. The FT-IR spectra of BT-rGO powder stay at air in different hours.

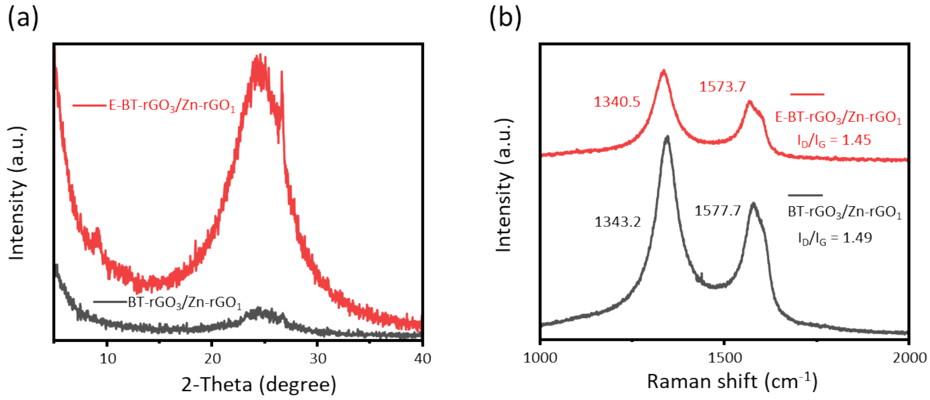

Figure S9. (a) XRD pattern, and (b) Raman spectra of BT-rGO_3/Zn-rGO_1 and E-BT-rGO_3/Zn-rGO_1.

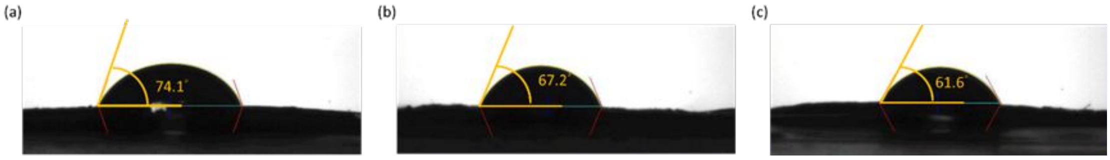

Figure S10. The contact angle of (a) E-BT-rGO_1/Zn-rGO_1, (b) E-BT-rGO_2/Zn-rGO_1, and (c) E-BT-rGO_3/Zn-rGO_1 electrodes.

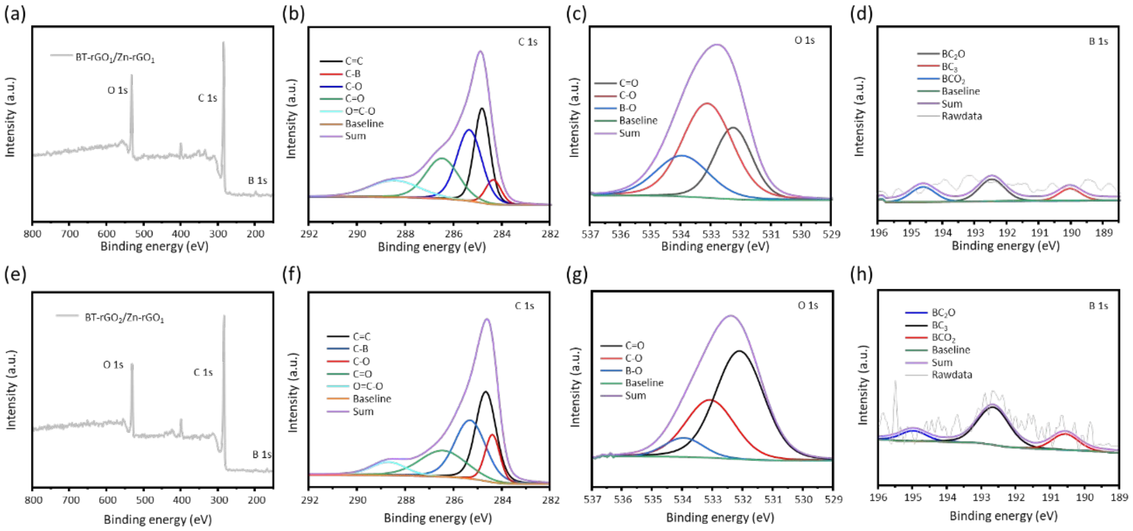

Figure S11. (a) The XPS spectra, and high-resolution spectra (b) C 1s (c) O 1s (d) B 1s of BT-rGO$_1$/Zn-rGO$_1$; (e) The XPS spectra, and high-resolution spectra (f) C 1s (g) O 1s (h) B 1s.

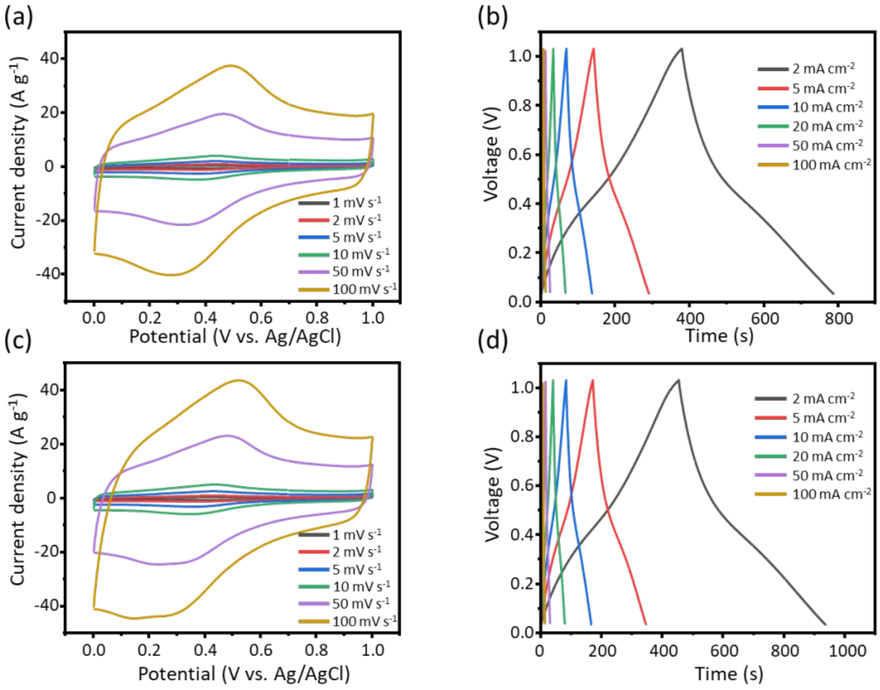

Figure S12. (a) CV curves at 1-100 mV s^{-1}, and (b) GCD curves at 2-100 mA cm^{-2} of E-BT-rGO$_1$/Zn-rGO$_1$; (c) CV curves at 1-100 mV s^{-1}, and (d) GCD curves at 2-100 mA cm^{-2} of E-BT-rGO$_2$/Zn-rGO$_1$.

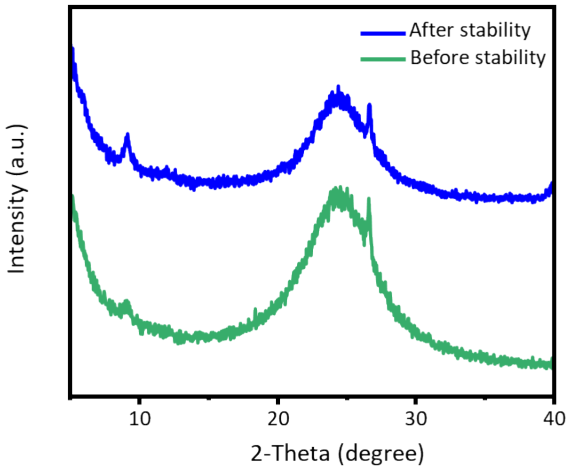

Figure S13. The XRD pattern of E-BT-rGO_3/Zn-rGO_1 before and after stability test.

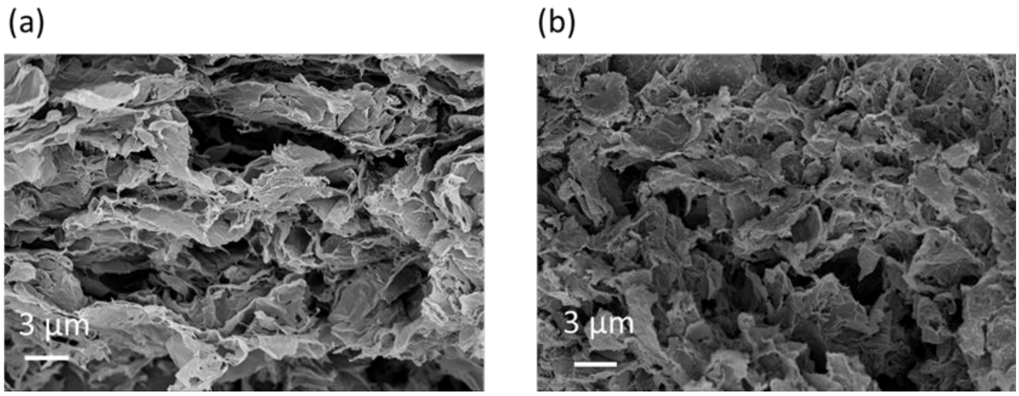

Figure S14. The SEM image of E-BT-rGO_3/Zn-rGO_1 electrode (a) before and (b) after 10,000 cycle stability test at 50 mA cm^{-2}.

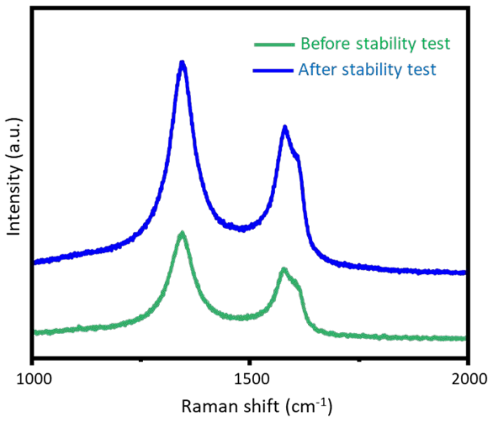

Figure S15. The Raman spectra of E-BT-rGO_3/Zn-rGO_1 electrode before and after 10,000 cycle stability test.

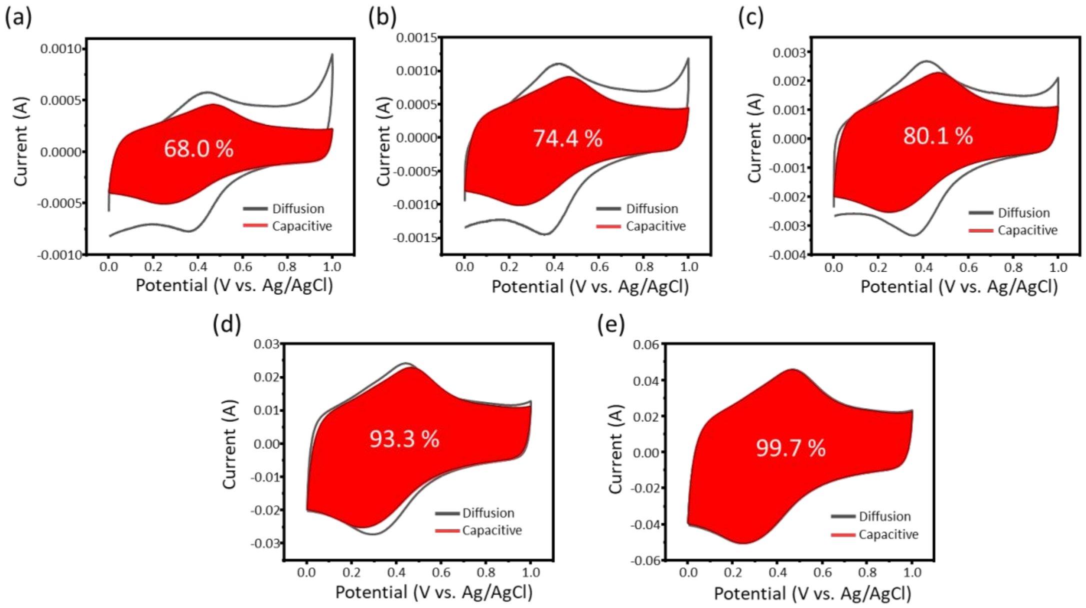

Figure S16. The separation of capacitive and diffusion contributions for E-BT-rGO_1/Zn-rGO_1 at (a) 1 mV s^{-1}, (b) 2 mV s^{-1}, (c) 5 mV s^{-1}, (d) 50 mV s^{-1}, and (e) 100 mV s^{-1}.

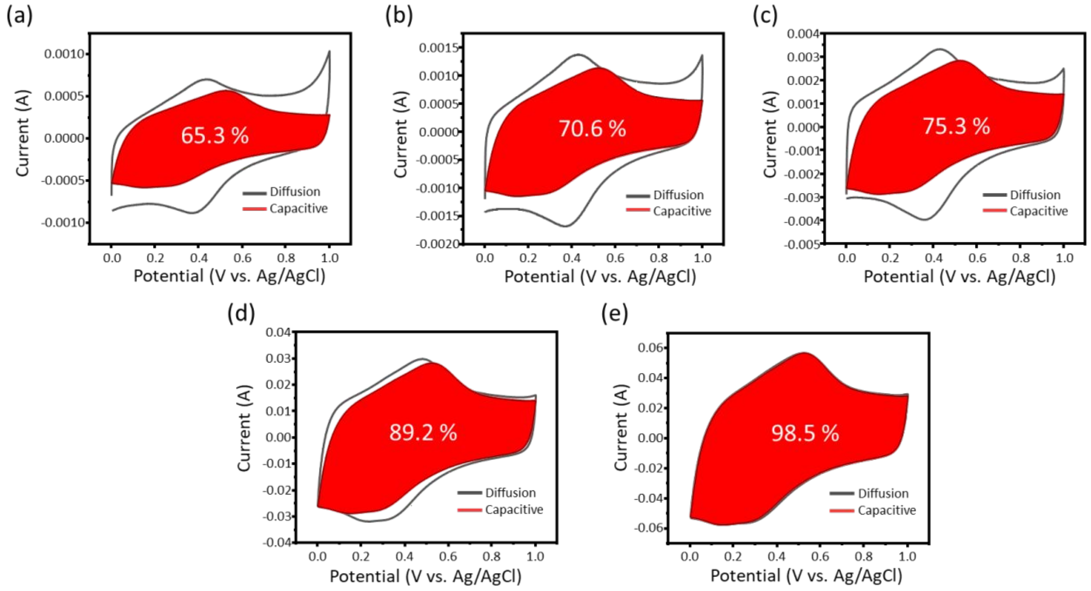

Figure S17. The separation of capacitive and diffusion contributions for E-BT-rGO_2/Zn-rGO_1 at (a) 1 mV s^{-1}, (b) 2 mV s^{-1}, (c) 5 mV s^{-1}, (d) 50 mV s^{-1}, and (e) 100 mV s^{-1}.

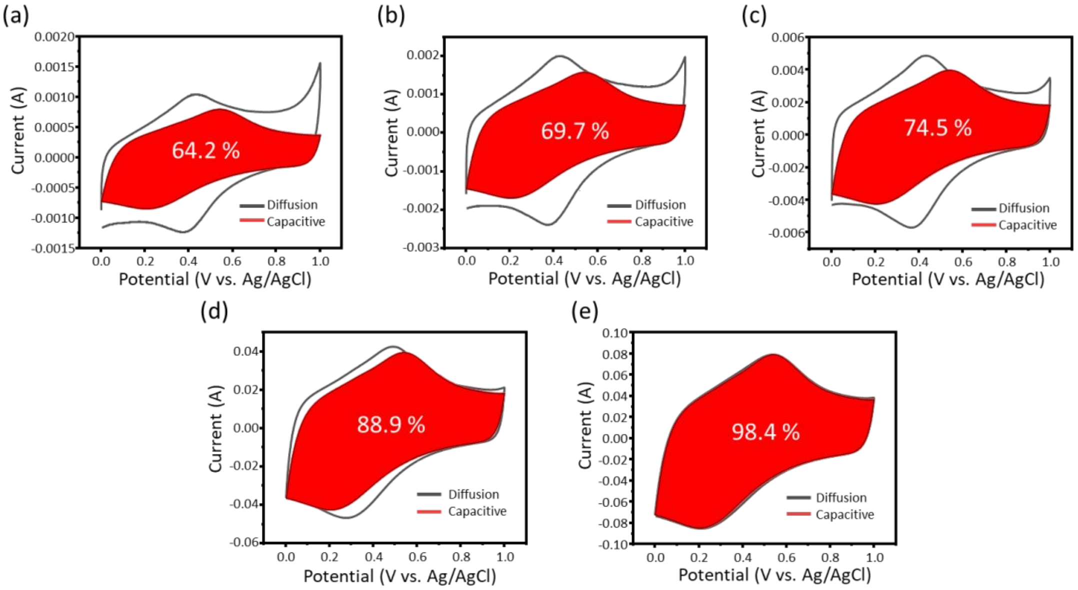

Figure S18. The separation of capacitive and diffusion contributions for E-BT-rGO_3/Zn-rGO_1 at (a) 1 mV s^{-1}, (b) 2 mV s^{-1}, (c) 5 mV s^{-1}, (d) 50 mV s^{-1}, and (e) 100 mV

s^{-1}.

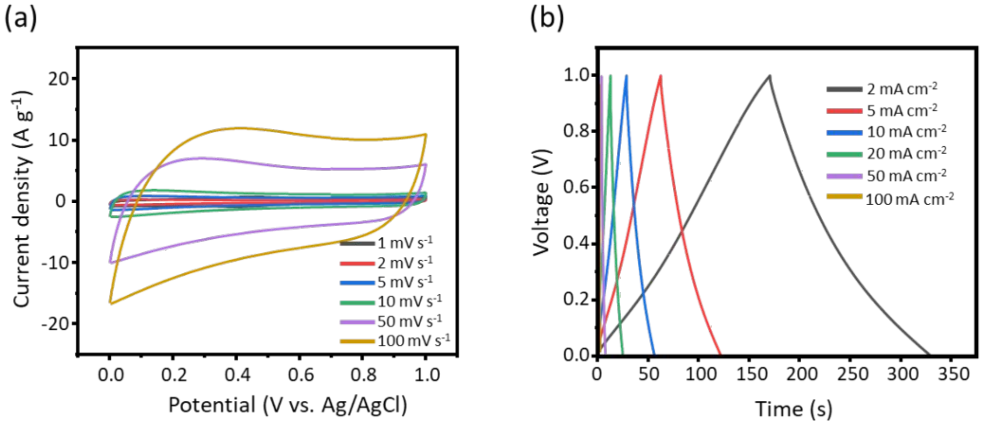

Figure S19. (a) CV curves at 1-100 mV s^{-1}, and (b) GCD curves at 2-100 mA cm^{-2} of the E-BT-rGO_3/Zn-rGO_1 electrode in two-electrode system in 1 M H_2SO_4.

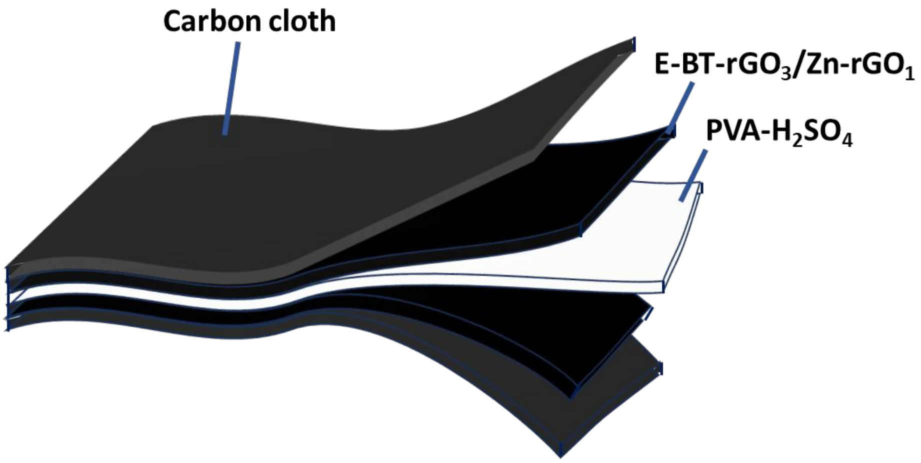

Figure S20. Schematic illustration of the E-BT-rGO_3/Zn-rGO_1 flexible solid-state supercapacitor device.

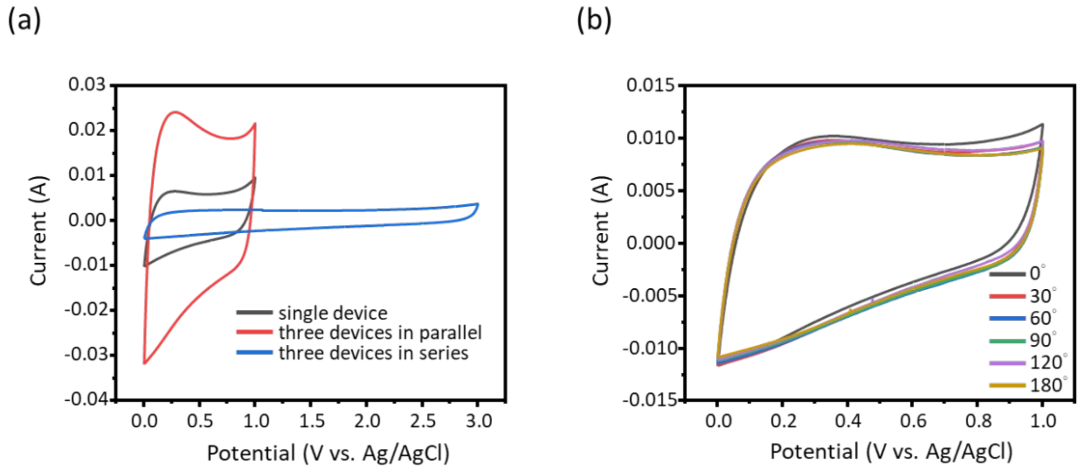

Figure S21. (a) CV curves of three series-connected and three parallel-connected devices at 50 mV cm^{-1}, and (b) CV curves at different bending angles at 50 mV s^{-1}

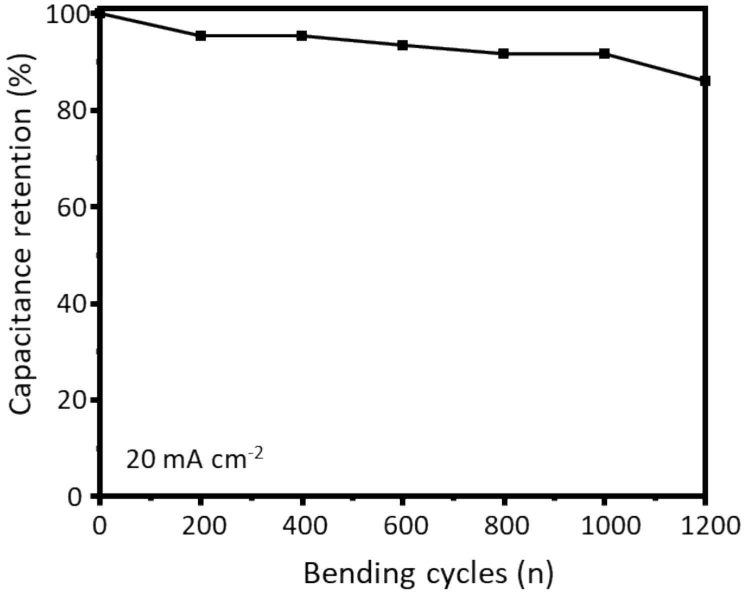

Figure S22. Effect of bending cycles on the capacitance retention of the E-BT-rGO$_3$/Zn-rGO$_1$ electrode.

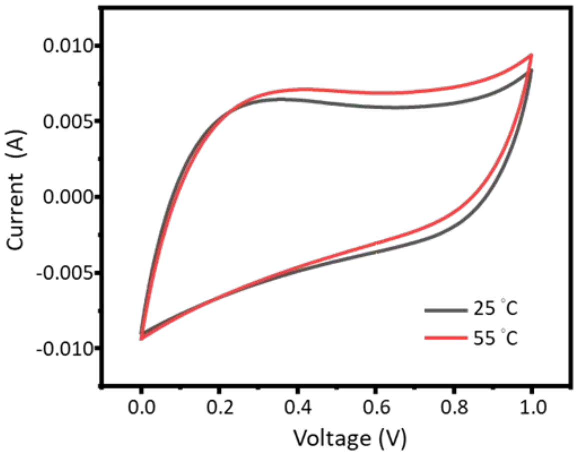

Figure S23. Effect of temperature on the specific capacitance of the E-BT-rGO_3/Zn-rGO_1 electrode at 50 mV s^{-1}.

Table S1. The elemental content of T-rGO and BT-rGO powder by XPS

Sample	C at %	O at %	B at %
T-rGO	76.4	23.6	0
BT-rGO	72.7	23.6	3.7

Table S2. The elemental content of BT-rGO_x/Zn-rGO_1 (x = 1, 2, and 3) and E-BT-rGO_3/Zn-rGO_1 by XPS

Sample	C at %	O at %	B at %
BT-rGO_1/Zn-rGO_1	84.2	15.4	0.4
BT-rGO_2/Zn-rGO_1	83.4	15.7	0.9
BT-rGO_3/Zn-rGO_1	83.8	15.3	1.0

Table S3. The parameter of BT-rGO_x /Zn-rGO_1 (x = 1, 2, and 3) electrodes.

Sample	BT-rGO:GO (mg)	Mass loading (mg cm^{-2})
BT-rGO_1/Zn-rGO_1	120:120	2.7±0.1
BT-rGO_2/Zn-rGO_1	160:80	2. 7±0.1
BT-rGO_3/Zn-rGO_1	180:60	2.8±0.2

Table S4. Comparison of boron-doped carbon material.

Material	Boron source	Specific capacitance	Ref.
BNMPC	ammonium borate	270 F g^{-1} at 0.5 A g^{-1}	(1)
NTGH-75	ammonium fluoroborate	268.8 F g^{-1} at 0.3 A g^{-1}	(2)
B-L/GCAs	boric acid	228 F g^{-1} at 0.5 A g^{-1}	(3)
PTh:B-rGO	boric acid	327.6 F g^{-1} at 0.5 A g^{-1}	(4)
BNG5	boric acid	336.76 F g^{-1} at 0.28 A g^{-1}	(5)
N_2B_{10}PC-800	boric acid	330 F g^{-1} at 0.2 A g^{-1}	(6)
BSNC-1.0	boric acid	233.5 F g^{-1} at 0.5 A g^{-1}	(7)
BNC-850	boric acid	341.5 F g^{-1} at 0.5 A g^{-1}	(8)
BNC-800	4-Fluorophenylboronic acid	258.5 F g^{-1} at 0.5 A g^{-1}	(9)
NBCs	boric acid	281 F g^{-1} at 0.2 A g^{-1}	(10)
BT-rGO_1/Zn-rGO_1	boric acid	323.31 F g^{-1} at 0.77 A g^{-1}	*this work*
BT-rGO_2/Zn-rGO_1	boric acid	372.46 F g^{-1} at 0.77 A g^{-1}	*this work*
BT-rGO_3/Zn-rGO_1	boric acid	443.63 F g^{-1} at 0.63 A g^{-1}	*this work*

References

(1) Guo, H.; Fu, H.; Lian, Y.; Zhao, J.; Zhang, H. B, N-Codoped Carbon Skeletons with Multistage Pore Structure for Supercapacitor and Capacitive Deionization. *Desalination* **2025**, *609*, 118862.

(2) Yu, T.; Zhou, Q.; Chen, J.; Ma, W.; Wang, C.; Fan, S.; Zhang, Y. The Synthesis of Nanocellulose/B, N, F Tri-Doped Graphene Composite Hydrogels for Supercapacitor Applications. *Vacuum* **2024**, *222*, 113036.

(3) Zhang, J.; Feng, Z.; Wang, S.; Cao, Z.; Qi, X.; Wang, B.; Xin, M.; Tang, J. Enhancing Electrochemical Performance of Lignin-Based Carbon Fibrous Aerogels Via Boron Doping. *Int. J. Biol. Macromol.* **2025**, *295*, 139639.

(4) Mahato, N.; Farooq, O.; Mahato, M. N.; Pradhan, S.; Kim, S.-J.; Wahid, M. A.; Mehta, Y.; Yoo, K.; Kim, J. Polycrystalline Polythiophene-Boron Doped Rgo Composite Exhibiting a Unique Charge Storage Mechanism and Exceptional Stability: A Rigorous EIS Investigative Model to Explore the Material's Integrity. *Appl. Surf. Sci.* **2025**, *711*, 164082.

(5) Kaur, A.; Pandey, O.; Brar, L. K. Synergic Effect of B and N Dopants in Graphene for Supercapacitance and Electrochemical Sensing Applications. *J. Phys. Chem. Solids* **2023**, *180*, 111460.

(6) Liu, Z.; Cui, X.; Yang, X.; Jiang, W.; Yuan, Z.; Wan, J.; Liu, Y.; Ma, F. Green Synthesis of N/B Co-Doped Layered Porous Carbon with High Gravimetric and Volumetric Capacitance for Supercapacitor. *J. Power Sources* **2025**, *630*, 236118.

(7) Bahadur, R.; Wijerathne, B.; Vinu, A. Multiple Heteroatom Doped Nanoporous Biocarbon for Supercapacitor and Zinc-Ion Capacitor. *ChemSusChem* **2024**, *17* (24), e202400999.

(8) Huang, J.; Peng, J.; Zeng, J.; Zheng, L.; Chen, H. B/N Co-Doped Porous Carbon Nanosheets with High B/N Doping Contents and Excellent Supercapacitor Performance. *J. Energy Storage* **2024**, *87*, 111514.

(9) Peng, J.; Dai, X.; Huang, J.; Zeng, J.; Zheng, L.; Chen, H. High-Yield Preparation of B/N Co-Doped Porous Carbon Nanosheets from a Cross-Linked Boronate Polymer for Supercapacitor Applications. *J. Energy Storage* **2023**, *59*, 106498.

(10) Xu, Z.; Wu, Z.; Chi, J.; Liu, Y.; Yin, Y.; Yang, Z.; Ma, C.; Li, W.; Luo, S.; Liu, S. Soft-Template Hydrothermal Synthesis of N and B Co-Doped Walnut-Shaped Porous Carbon Spheres with Hydrophilic Surfaces for Supercapacitors. *Appl. Surf. Sci.* **2023**, *638*, 158016.
